# Genotyping of *Mycobacterium leprae* for better understanding of leprosy transmission in Fortaleza, Northeastern Brazil

**DOI:** 10.1371/journal.pntd.0006117

**Published:** 2017-12-15

**Authors:** Amanda N. B. Fontes, Luana N. G. C. Lima, Rosa M. S. Mota, Rosa L. F. Almeida, Maria A. Pontes, Heitor de S. Gonçalves, Cristiane C. Frota, Varalakshmi D. Vissa, Patrick J. Brennan, Ricardo J. P. S. Guimaraes, Carl Kendall, Ligia R. F. S. Kerr, Philip N. Suffys

**Affiliations:** 1 Laboratory of Molecular Biology Applied to Mycobacteria, Oswaldo Cruz Institute, Rio de Janeiro, Brazil; 2 Department of Pathology, State Health Office, Fortaleza, Brazil; 3 Department of Statistics and Applied Mathematics, Federal University of Ceará, Fortaleza, Brazil; 4 Post Graduation Program of Public Health, University of Fortaleza, Fortaleza, CE, Brazil; 5 Reference Center on Dermatology Dona Libânia, State Health Office, Fortaleza, Brazil; 6 Department of Pathology, Federal University of Ceará, Fortaleza, Brazil; 7 Department of Microbiology, Immunology and Pathology, Colorado State University, Fort Collins, Colorado, United States; 8 Laboratory of Geoprocessing, Evandro Chagas Institute, Belém, Brazil; 9 Department of Global Community Health and Behavioral Sciences, Tulane School of Public Health and Tropical Medicine, New Orleans, United States; 10 Department of Community Health, College of Medicine, Federal University of Ceará, Fortaleza, CE, Brazil; 11 Department of Biomedical Sciences, Mycobacteriology Unit, Tropical Institute of Medicine, Antwerp, Belgium; Yale University Yale School of Public Health, UNITED STATES

## Abstract

Leprosy is endemic in large part of Brazil with 28,761 new patients in 2015, the second largest number worldwide and reaches 9/10.000 in highly endemic regions and 2.7/10.000 in the city of Fortaleza, Ceará, Northeast Brazil. For better understanding of risk factors for leprosy transmission, we conducted an epidemiologic study supplemented by 17 locus VNTR and SNP 1–4 typing of *Mycobacterium leprae* in skin biopsy samples from new multibacillary (MB) patients diagnosed at a reference center in 2009 and 2010. Among the 1,519 new patients detected during the study period, 998 (65.7%) were MB and we performed DNA extraction and genotyping on 160 skin biopsy samples, resulting in 159 (16%) good multilocus VNTR types. Thirty-eight of these patients also provided VNTR types from *M*. *leprae* in nasal swabs. The SNP-Type was obtained for 157 patients and 87% were of type 4. Upon consideration all VNTR markers, 156 different genotypes and three pairs with identical genotypes were observed; no epidemiologic relation could be observed between individuals in these pairs. Considerable variability in differentiating index (DI) was observed between the different markers and the four with highest DI [(AT)15, (TA)18, (AT)17 and (GAA)21] frequently demonstrated differences in copy number when comparing genotypes from both type of samples. Excluding these markers from analysis resulted in 83 genotypes, 20 of which included 96 of the patients (60.3%). These clusters were composed of two (n = 8), three (n = 6), four (n = 1), five (n = 2), six (n = 1), 19 (n = 1) and 23 (n = 23) individuals and suggests that recent transmission is contributing to the maintenance of leprosy in Fortaleza. When comparing epidemiological and clinical variables among patients within clustered or with unique *M*. *leprae* genotypes, a positive bacterial index in skin biopsies and knowledge of working with someone with the disease were significantly associated with clustering. A tendency to belong to a cluster was observed with later notification of disease (mean value of 3.4 months) and having disability grade 2. A tendency for lack of clustering was observed for patients who reported to have lived with another leprosy case but this might be due to lack of inclusion of household contacts in the study. Although clusters were spread over the city, kernel analysis revealed that some of the patients belonging to the two major clusters were spatially related to some neighborhoods that report poverty and high disease incidence in children. Finally, inclusion of genotypes from nasal swabs might be warranted. A major limitation of the study is that sample size of 160 patients from a two year period represents only 15% of the new patients and this could have weakened statistical outcomes. This is the first molecular epidemiology study of leprosy in Brazil and although the high clustering level suggests that recent transmission is the major cause of disease in Fortaleza; the existence of two large clusters needs further investigation.

## Author summary

Leprosy is a transmissible disease that is still endemic in several countries including in Brazil, a country with highly variable region associated incidence of disease. Fortaleza is a city in Northeast Brazil with high incidence and conventional epidemiology studies are suggestive for high levels of recent transmission. Genotyping of *M*. *leprae* allows the recognition of individuals that have been infected with the same strain (called a cluster) and therefore being suggestive for belonging to the same transmission network. In the present work, by analyzing genotypes of *M*. *leprae* in the skin lesion of multibacillary patients, we made observations that improve our knowledge on interpretation of genotypes and clusters and confirm the high levels of recent transmission in the city of Fortaleza. This is one of the few studies that used molecular epidemiology to look for risk factors for recent transmission of leprosy and to our knowledge, the first in Brazil. Our data support further investigation of the workplace as a source of infection, preferentially by a study designed on a larger number of patients and including analysis of *M*. *leprae* present in the nose.

## Introduction

Leprosy, caused by infection with *Mycobacterium leprae* remains a significant public health problem in many developing countries. The disease presents a wide spectrum of clinicopathologic forms that ranges from tuberculoid leprosy (TT) to borderline forms and lepromatous leprosy (LL) and lesions involve skin and peripheral nerves. Disease can be paucibacillary (PB) or multibacillary (MB) with the most severe LL form involving organs such as liver, spleen and bone marrow and the bacterial burden in such patients is massive and causes severe deformities when not treated. Multi-drug therapy using dapsone, rifampicin and clofazimine was implemented in the 1980s and has considerably reduced disease prevalence, but that is not the case with incidence, implying that leprosy is still being transmitted to a considerable extent [1]

As *M*. *leprae* cannot be cultured on artificial media, molecular techniques have been used for better characterization of the organism [2, 3, 4, 5], including the deciphering of the genome sequence [6]. Single nucleotide polymorphisms (SNPs) analysis allowed studies on phylogeography of leprosy, evolving models for the global spread of *M*. *leprae* [7,8]. Besides SNPs, Variable Number Tandem Repeats (VNTRs) are used for genotyping and a certain relation between the number of certain VNTR alleles and SNP-Type has been observed [9], showing that VNTR typing adds to our knowledge on spread of leprosy. Multiple-locus variable number tandem repeats analysis (MLVA) of a set of micro- and mini-satellites of *M*. *leprae* is a fingerprinting procedure for differentiation at the strain level [10, 11, 12, 13] and useful during transmission studies, to distinguish reactivation from re-infection [14] and to study bacterial population structure on different levels and countries, as described for Brazil [15,16], China [9, 17, 18, 19], India [20, 21, 22, 23, 24], Philippines [25, 26], Thailand [27, 28], Mexico [29], Colombia [30, 31] and the United States [32].

Fortaleza is the capital of Ceará, a state located in northeastern Brazil. In 2015, 80.5% of the 184 municipalities in the state diagnosed new patients of leprosy and 10% were classified as being hyperendemic, defined by having an incidence of higher that 4/10.000. Ceará is one of the poorest regions of the country, reporting 1.743 new leprosy patients in the same year, including 528 in the capital, representing an incidence rate of 2.7/10,000 inhabitants. MB is detected in two thirds of these patients and 5.9% of the total patients reported in the state are younger than 15 years of age, both of which are indicators of ongoing and recent transmission seems of the disease [33].

Previous genotyping of *M*. *leprae* strains in Brazil, from a set of unrelated patients from the Southeast region of the country demonstrated a high VNTR based genetic variability in predomintly SNP-Type 3 background [15]. Later, it was observed that SNP-Type 4 is much more frequent in the North-northeast part of the country [6]. Although preliminary data on use of genotyping to add to transmission studies have been presented in Ceará [34], Mato Grosso [35] and Pará [36], no full reports exist on molecular epidemiology studies of leprosy and the rearch for risk factors for recent transmission in Brazil; therefore our study addresses this gap.

## Materials and methods

### Setting

Fortaleza is the capital and also largest city of the state of Ceará, and the fifth largest city (314,930 km^2^) in Brazil with 2,627,482 inhabitants in 2017. It has 120 neighborhoods and the highest population density among the country's capitals. Although Fortaleza has the tenth highest GDP in the country and the highest in the Northeast region, it has the typical uneven distribution of wealth observed in most of Brazil’s major cities. Besides being an important industrial and commercial center, it is the second most desired tourist destination in Brazil and fourth in number of visitors [37, 38].

### Study design

This study was designed to better understand the clinical and epidemiological characteristics of leprosy in the city of Fortaleza. A cross-sectional study was conducted from November 2008 to December 2010 and during this period, all new leprosy patients diagnosed by trained dermatologists of the National Reference Center of Dermatology Dona Libânia (CDERM) were invited to participate in the study. This tertiary reference center serves about 80% of the almost 800 new leprosy patients diagnosed annually in Fortaleza and is the most important reference center for skin disease, including leprosy, in that city [39].

Patients were diagnosed by clinical evaluation; microscopic evaluation of bacillary index of acid fast bacteria in slit skin smears (SSS) analysis and histopathological evaluation of biopsy specimens. Patients were classified according to Ridley-Jopling criteria based on histological study and bacterial indices (BI) [40]. All new patients responded to a detailed questionnaire that included demographic, epidemiologic, socioeconomic, environmental and behavioral components. In addition to the questionnaire, data for the patients were introduced and maintained by registered health workers in the SINAN database (http://portalsinan.saude.gov.br). A second skin biopsy and nasal swab was collected for genotyping of *M*. *leprae* in a subset of all diagnosed patients.

### Specimen collection and DNA extraction

The skin biopsy samples were collected using a 5 mm punch. Tissue for histopathology was treated with formol and embedded in paraffin while the tissue for genotyping was placed in a sterile 1.5 mL tube and stored at -20°C. The DNA was extracted by using the DNeasy Blood & Tissue kit (Qiagen Biotecnologia do Brasil Ltda, SP, Brazil) following the manufacturer's guidelines.

Nasal swabs were collected from patients who also provided a second skin biopsy for genotyping, by gently rubbing a swab previously wetted with Tris-EDTA buffer (pH 8.0), in one side of each nostril over the lateral conchae. After collection, each swab was immersed in a sterile and labeled tube and stored at -20°C until processing as described by Lima et al. [41]

### Genotyping

Genotyping by MLVA of 17 VNTRs was performed as described by Kimura et al. [13] and based on four multiplex PCRs that generated 17 amplicons. The allele for each VNTR locus is the copy number of the repeats which was determined by denaturation of amplicons and capillary gel electrophoresis on the sequencer ABI 3130 Genetic Analyzer, using the internal molecular weight sizing standards (LIZ 500). The copy number of each locus was calculated based on the size of the PCR amplicon using the Peak Scanner software (Applied Biosystems do Brasil) and comparing to previously calibrated *M*. *leprae* strain NHDP63. To study reproducibility of the assay, DNA from five *M*. *leprae* samples from Brazil was sent to CSU for comparative analysis of the alleles.

For differentiation of four genotypes of *M*. *leprae* based on three SNPs, we used a procedure that combined PCR-restriction enzyme analysis (REA) and direct sequencing as described by Sakamuri et al. [26]. Differentiation of genotypes 1/2 from 3/4 was obtained by submitting to *Bst*UI mediated PCR-RFLP analysis of the locus at nucleotide position 2,935,685; digestion occurs in case of genotype 3/4 and lack of digestion for genotype 1/2. Differentiation of genotypes 3 and 4 is obtained by *Sml*I mediated PCR-RFLP at nucleotide position 14,676; digestion indicates SNP-Type 4 and lack of SNP-Type 3. Differentiation of SNP-Type 1 or 2 was performed by direct PCR sequencing as described by Monot et al. [7].

### Cluster definition and genotype comparison

The copy number of all alleles were introduced into Microsoft Excel files and imported into Bionumerics software (version 7.6; Applied Maths; Sint Martens Latem, Belgium).

Definition of clustering was based on comparison of the copy number of the VNTRs using two different stringencies: either considering those that presented identical copy number for all 17 alleles, or considering those that had identical copy number in 13 alleles, excluding the four most variable loci. A similarity matrix was constructed using the categorical similarity coefficient and the unweighted pair group method with arithmetic mean (UPGMA). This was the basis for a complete linkage tree, a circular top score UPGMA tree and a range of minimum spanning trees (MST).

### Spatial analysis

**Fig 1:**
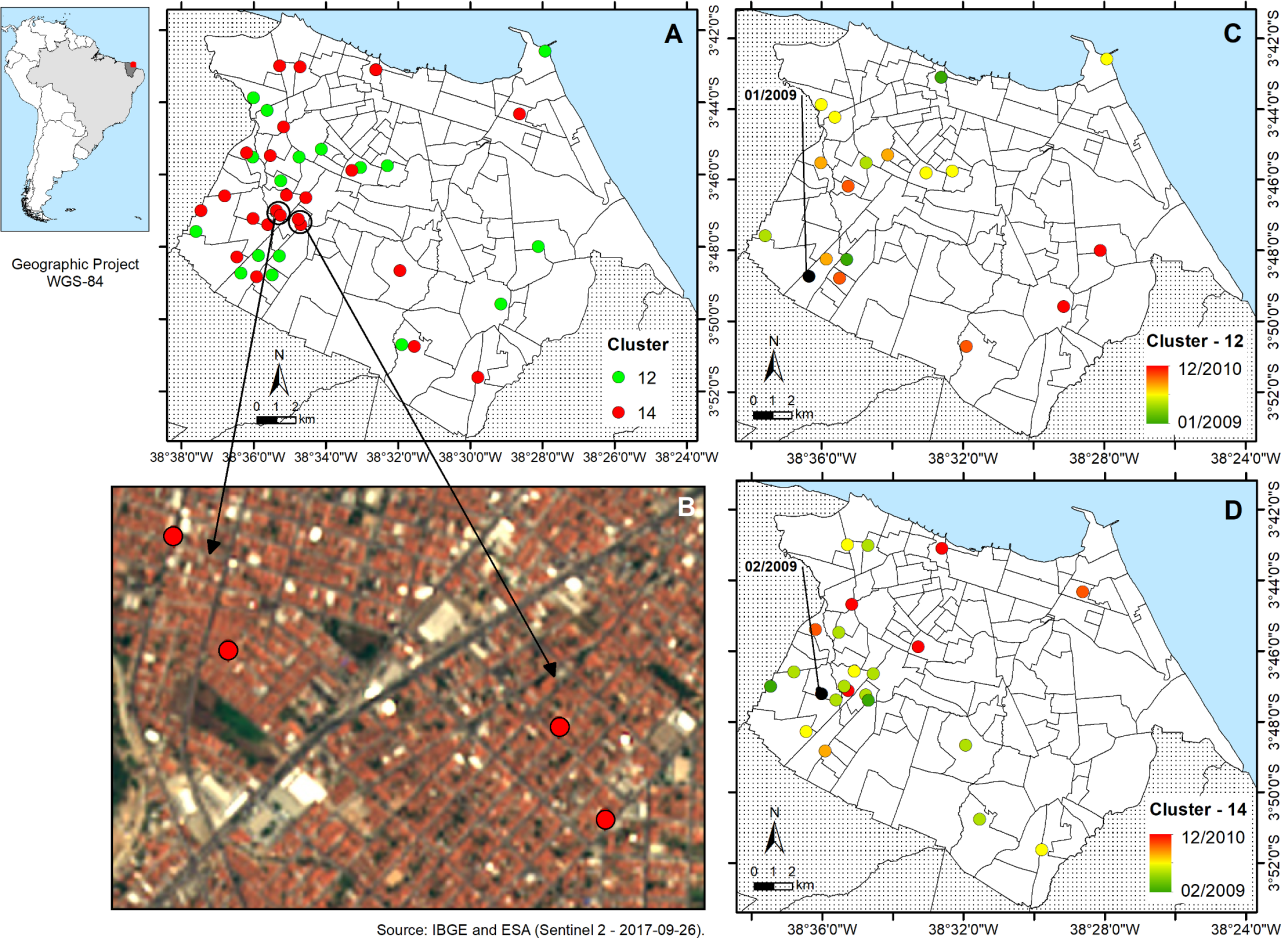
Geographic position of the 19 and 23 patients from respectively cluster 12 and 14 in Fortaleza, State of Ceará. Map of Fortaleza (A), details from the region with two clustered pairs (B), the upper pair is localized in Bonsucesso and the lower in Vila Peri. Space/time distribution of clusters 12 (C) and 14 (D); the highlight in black refers to the first case for each cluster.

The cartographic bases and the population used were obtained from the Brazilian Institute of Geography and Statistics (http://www.ibge.gov.br/). The coordinates were obtained using a global positioning system (GPS) and stored in a geographic database (BDGeo). Data were used to generate graphics, satellite imagery processing, to establish topological relations between the graphic elements and their attributes, spatial analysis and visualization through thematic maps. We evaluated the spatial analysis Kernel density estimation (KDE) using a fixed radius of 2 km. Analyses were performed in ArcGis (http://www.esri.com/) and TerraView (http://www.dpi.inpe.br/menu/Projetos/terraview.php). In TerraView it was possible to build a dual Kernel or Kernel ratio, based on the number of patients and the population [42]. We used the interpolator points by Inverse Distance Weighting (IDW) to estimate the cell values using a weighted linear combination of a set of sampling points. The satellite image in Fig 1 was generated using the sensor Sentinel 2 of the European Space Agency (ESA) (https://sentinel.esa.int/web/sentinel/user-guides/sentinel-2-msi) with Open Access CC-BY License (http://open.esa.int/).

### Statistical analysis

For evaluation of the association of the demographic, clinical and environmental/behavior variables and having a clustered or a unique *M*. *leprae* genotype, chi squared and Fisher exact tests were used. Mann-WhitneyU test was used for evaluation of differences between a single characteristic in individuals with clustered genotypes or unique patterns.

### Ethical considerations

An informed consent form was signed by the participants of the study, authorizing the collection of clinical samples. The present study was approved by the Ethics Committee of CDERM and the national ethical committee.

## Results

### Sampling and patients and data

At CDERM, 830 (284 PB and 546 MB) and 689 (237 PB and 452 MB) new leprosy patients were diagnosed respectively in 2009 in 2010, totaling 1519 in the study period and among these, 998 were MB patients (65.7%). Recruitment was conducted only on two days per week, which was further reduced in December, January and July and on holidays. This resulted in the collection of a second biopsy specimen for genotyping from 301 MB patients only of whom we received 160 (only 92 from 2009 and 68 from 2010). This resulted in *M*. *leprae* genotyping of 16.8% and 15% of the newly diagnosed MB patients respectively in 2009 and 2010. From these 160 patients, 101 also had nasal swab collected.

Because the questionnaire was developed for a larger case-control study evaluating risk factors for leprosy and the patients within the study presented here only partially overlapped with the larger study, we accessed data from the SINAN database (http://portalsinan.saude.gov.br) for 61 of the 159 patients (31%).

### Genotyping

Of the 160 patients biopsies submitted to *M*. *leprae* genotyping, 159 yielded high quality MLVA-based and 157 SNP-based genotypes and are presented in **S1 Table** and **S1 Fig**. Initially, 134 *M*. *leprae* were defined as SNP-Type 4 (85%), 15 as SNP-Type 3, three as SNP-Type 1 and six samples could be characterized only to the SNP-Type 1 or 2 level because of insufficient material for sequencing. Four isolates with SNP-Type 3 were grouped within the MLVA-based clusters of isolates with SNP type 4 so we suspected wrong classification due to partial digestion during PCR-RFLP. Two samples had sufficient material left to repeat and both were indeed confirmed as being SNP-Type 4, resulting in 136 SNP-Type 4 (86%) and 13 (8.2%) SNP-Type 3.

The MLVA-based typing results are presented in **S1 Table** and all but eight strains yielded the complete 17 locus-based genotypes (95%), five isolates failed in the amplification of one locus while another two lacked alleles for five and six alleles respectively. The latter strain was clustered with another but not included in the analysis of clusters at high cluster stringency. Two isolates presented a double peak, one at (TA)18 and another at (AT)15 and these alleles were not considered for analysis. The differentiating power and allele distribution of the satellites is presented in **Table 1**, and varied between 0 and 0.93, and 1 and 22, respectively. Three markers–[(GGT)5, 6–3 and 21–3] were invariable, four [(AT)15, (TA)18, (AT)17 and (GAA)21] were highly discriminatory with a Hunter-Gaston discriminatory index (HGDI) above 0.8, and the rest had a HGDI of less than 0.8. Interestingly, the copy number distribution pattern of 18–8 is different from that of the other markers and presented a bimodal pattern (**Table 1)**.

**Table 1:** Allele distribution and simpson index among MLVA based genotypes and SNP-types.

Genetic Marker																		
Copy number	(AC)8b	(GTA)9	(GGT)5	(AT)17	6–3	21–3	(AC)9	(AT)15	(AC)8a	27–5	6–7	(TA)18	(GAA)21	(TA)10	23–3	12–5	18–8	SNP
**1**															12			3
**2**						157				3					147		1	
**3**					159					1						2	139	13
**4**			158							6						134		136
**5**										148	2	1				20		
**6**	2	1					1			1	134							
**7**	94	2					89				16						5	
**8**	63	15					62		77		4			120			12	
**9**		47					7		63		1		2	20			1	
**10**		55		10					14		1	1	11	9				
**11**		19		17					5			2	10	4				
**12**		16		22				2				2	41	1				
**13**		1		41				6				1	29	1				
**14**				18				12				9	29					
**15**		2		8				8				12	14					
**16**				11				25				17	7					
**17**				8				10				20	6					
**18**				6				9				12	3					
**19**				6				11				17	3					
**20**				6				11				8	1					
**21**				2				9				9	1					
**22**				1				8				5						
**23**				1				10				7						
**24**								6				1						
**25**				1				7				12						
**26**								5				12						
**27**								1										
**28**								6				3						
**29**								2				1						
**30**								2				3						
**31**												2						
**32**								1										
**33**								1										
**34**								1										
**Doubdful**								1				1						6 (1/2)
**ND**		1	1	1		2		5			1	1	2	4		3	1	
**Number of alleles**	3	9	1	15	1	1	4	22	4	5	6	22	13	6	2	3	5	
**Simpson's index**	**0.496**	**0.761**	**0**	**0.876**	**0**	**0**	**0.536**	**0.934**	**0.604**	**0.133**	**0.271**	**0.929**	**0.848**	**0.382**	**0.140**	**0.247**	**0.221**	**0.214**

ND = not done

Regarding cluster analysis, when using the highest stringency including 17 markers, we observed 157 different genotypes formed by 154 singletons and three clusters of two patients each, resulting in an overall cluster level of 3.8% (6/157). Upon analysis of the data of the patients within each of the three clusters and or of those with unique genotypes, no particular risk factor for belonging to one of or any cluster was identified.

However, when excluding the four markers with HGDI > 0.8, 83 different genotypes were detected, 63 unique ones and another 20 found in 96 patients, resulting in a cluster rate of 60.4% (96/159). The two largest clusters were composed of 23 and 19 patients and the remaining clusters were composed of one of six, two of five, one of four, six of three and eight of two patients. Again, no clear patient characteristic was detected that could explain the formation of an individual cluster but when analyzing the data of those belonging to a genotype cluster or not, some significant associations and tendencies for clustering were observed. We observed a significant association of clustering being BI positive (p = 0.037) or having worked (p = 0.049) with someone with leprosy (note: working together has p = 0.25). Surprisingly, the variable ‘having lived with someone who had leprosy’ demonstrated an inverse relation with clustering (p = 0.065). Although not significant, an association was observed between clustering and disability (p = 0.445), mainly because of a tendency to have more grade 2 disability among clustered patients (13.1% against 5.1%) and longer time between observing first lesion and diagnosis/disease notification (p = 0.14). Another unusual finding was that alcohol consumption was significantly associated with non-clustering, i.e., of having unique genotypes (p = 0.047) (**Table 2).** Note however that some of these associations occurred with the number of patients for some categories being < 5 and a detailed relation between clustering and variables is presented in **S2 Table**.

**Table 2:** Bivariate analysis of a selection of demographic, socioeconomic, behavioral, and environmental variables with leprosy genotype clustering.

Variables	Non clustered* Nr (%)	Clustered* Nr (%)	P value
**Number of months between observing the first lesion and diagnosis/notification**	62 (2.1)	92 (5.5)	0.14
**Acid Fast Bacilli**			
Pos	30 (49.2%)	60 (31%)	**0.037**
Neg	29 (50.8%)	27 (69%)	
**Disability**			
Grade 0	26 (66.7%)	38 (62.3%)	0.43
Grade 1	11 (28.2%)	15 (24.6%)	
Grade 2	2 (5.1%)	8 (13.1%)	
No info	10 (16.1%)	8 (8.5%)**	
**Alcohol use**			
Never	24 (58.5%)	44 (77.2%)	**0.047**
Max once a week	12 (29.2%)	12 (21.1%)	
Several times a week	5 (12.2%)	1 (1.8%)	
**How many people with leprosy you know?**	22 (1.82%)	23 (1.44%)	0.13
**Has this person lives or lived with you?**			
Yes	8 (36.4%)	3 (13%)	0.09
No	14 (63.6%)	20 (87%)	
**Is it presently a contact at work?**			
Yes	0	3 (11.1%)	0.25
No	21 (100%)	24 (88.9%)	
**Has it been a work contact in the past?**			
Yes	0	5 (21.7%)	0.049
No	22 (100%)	18 (78.3%)	

98 of the individuals had data generated as part of the project with prospective data; the other 61 (38.4%) had their data retrieved from the SINAN (http://portalsinan.saude.gov.br)

*Clustering defined by excluding the four most variable VNTRs

**not included for chi-square calculation

We also performed chi-square analysis of patients and other characteristics of the 18 patients from cluster 12 and 23 patients from cluster 14 (totalling 41 among 159 = 25.8% in cluster) and observed no significant association of the clustered cases when compared to the rest of any of the variables. Additionally, we plotted the date of diagnosis of the patients, clustered cases and those belonging to the two major clusters (12 and 14) on a monthly based time scale of the study period and although some higher frequency of diagnosis was observed between March and June of 2009, no particular independent increase in clustering was observed during the study period (**S2 Fig**).

Among 38 patients, MLVA patterns were also available from nasal swabs, with the exclusion of alleles 6–3 and 18–8, not performed in this sample type and as described recently, difference in copy number of the alleles with highest DI was observed in the *M*. *leprae* genotypes when comparing both samples in a considerable number of patients [43]. Upon inclusion of the genotypes of *M*. *leprae* present in nasal swabs in the analysis, we observed that eight genotypes from nasal swab were part of some cluster, increasing the number of clustered patients by 10. One cluster with a genotype shared by *M*. *leprae* in skin biopsy of five patients increased to eight when considering genotypes in nasal swab while three new clusters were observed, one composed of the genotype observed in the nasal swab of two patients, and two others composed of a genotype that was observed in the nasal swab and skin biopsy of two patients each (**S3 Table**).

### Spatial analysis

Data to perform spatial analysis was available for 156 patients and demonstrated clearly a higher density in the western part of Fortaleza (**S3A Fig).** The Figure also presents the number of patients per neighborhood (**S3B Fig**), the population estimated by the 2010 census by neighborhood (**S3C Fig**), KDE using georeferenced homes of patients (**S3D Fig**), KDE using neighborhood (**S3E Fig**) and dual Kernel using neighborhood (**S3F Fig**) in Fortaleza. The neighborhood with the highest number of patients is Granja Lisboa. The result obtained by using KDE in the neighborhoods showed, as expected, the same clusters, both centered in the neighborhoods Bom Jardim, Bonsucesso, Granja Lisboa and Granja Portugal (**S3D** and **S3E Fig**). However, when applying dual Kernel analysis, two clusters are observed, one including Granja Lisboa and Siqueira (southwest region), the same as observed using Kernel (neighborhood), and the second centered in the neighborhood Jacareacanga in northern Fortaleza.

**Fig 2:**
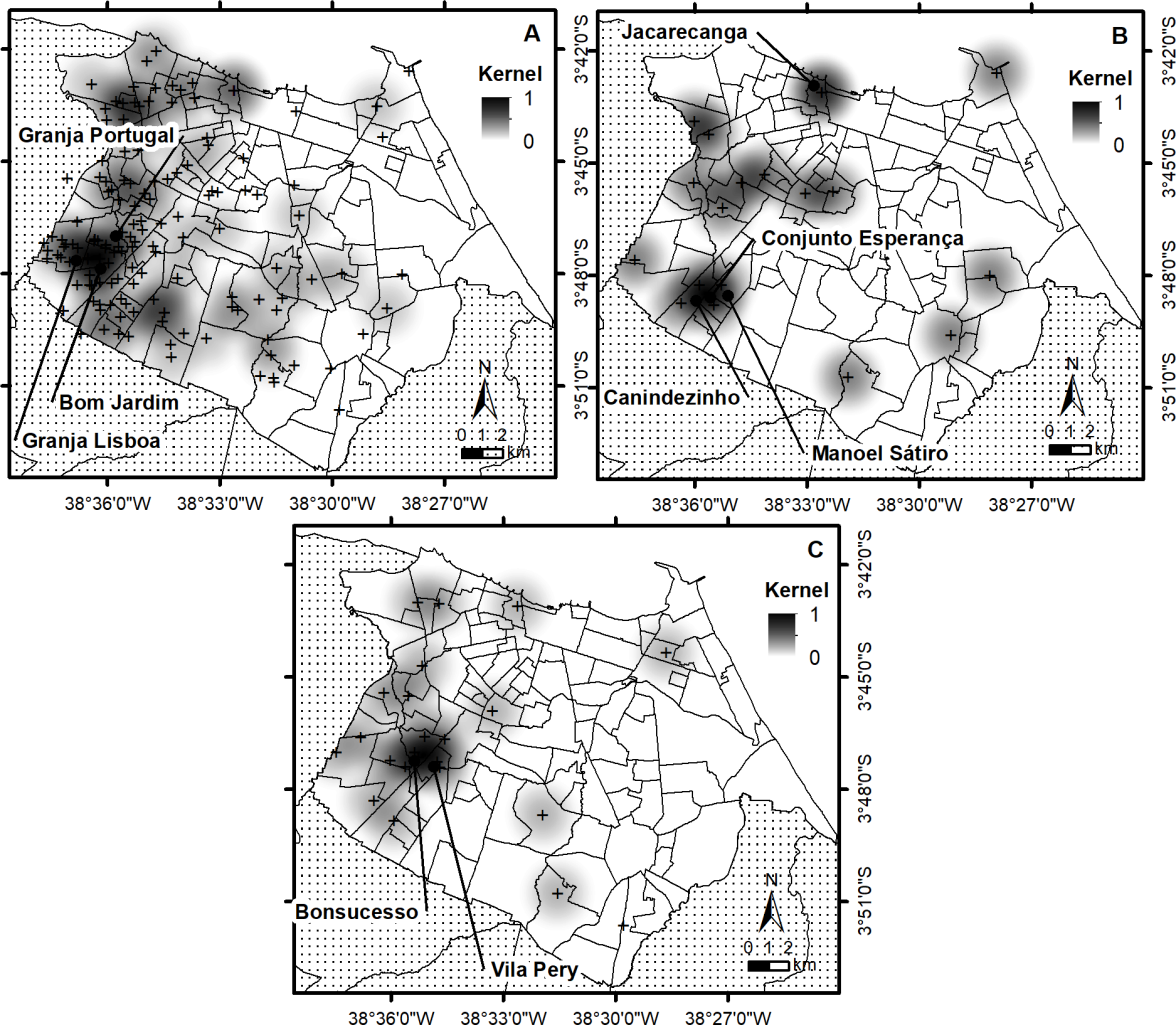
Spatial analysis and visualization through thematic maps after kernel density estimation (KDE) using a fixed radius of 2 km on all patients (A), to the 19 patients of cluster 12 (B) and the 23 patients of cluster 14 (C). The cross indicates the geographic position of the patients.

The distribution of the patients among the non-clustered patients and for each cluster across the neighborhoods of the city is presented in **S4 Table** and demonstrates that nine groups with clustered genotypes had at least two patients in the same neighborhood (groups 2, 7, 8, 9, 12, 14, 16, 17 and 19). Overall, patients with the same *M*. *leprae* genotype are spread across the city, except for the biggest cluster 14 showing two pairs of two very nearby patients (**Fig 1**). When performing KDE analysis using a distance of 2 km concentrating on the distribution of the patients from the two largest clusters, association was observed with some neighborhoods. The cluster formed by 19 patients was associated with Jacareacanga, Canindezinho, Conjunto Esperança and Manoel Sátiro, while those of the cluster with 23 isolates with Bonsucesso and Vila Pery (**Fig 2**). However, when performing the same type of analysis with the 62 patients with unique genotypes, we observed association with Granja Lisboa, Granja Portugal and Bom Jardim. Note however that the number of patients with unique patterns is about three times higher than those in each of the two biggest clusters.

Finally, we plotted distribution of patients and performed spatial analysis according the number of lesions and number of bacilli observed by bacilloscopy (**S4 Fig**). The number of lesions varied between 0 and 88 (total of 2120, medium value 13.6 and standard deviation of 13.2) and bacterial indices were between 0 and 6+ (total 421, median value 2.7 and SD 1.99). Although we observed that patients with high number of lesions or high bacillary load were spread over the city, two neighborhoods that were associated with cluster 12 (Canindezinho and Conjunto Esperança) and 14 (Bonsucesso) presented patients with high BAAR.

### Population structure and different clustering procedures

**Fig 3:**
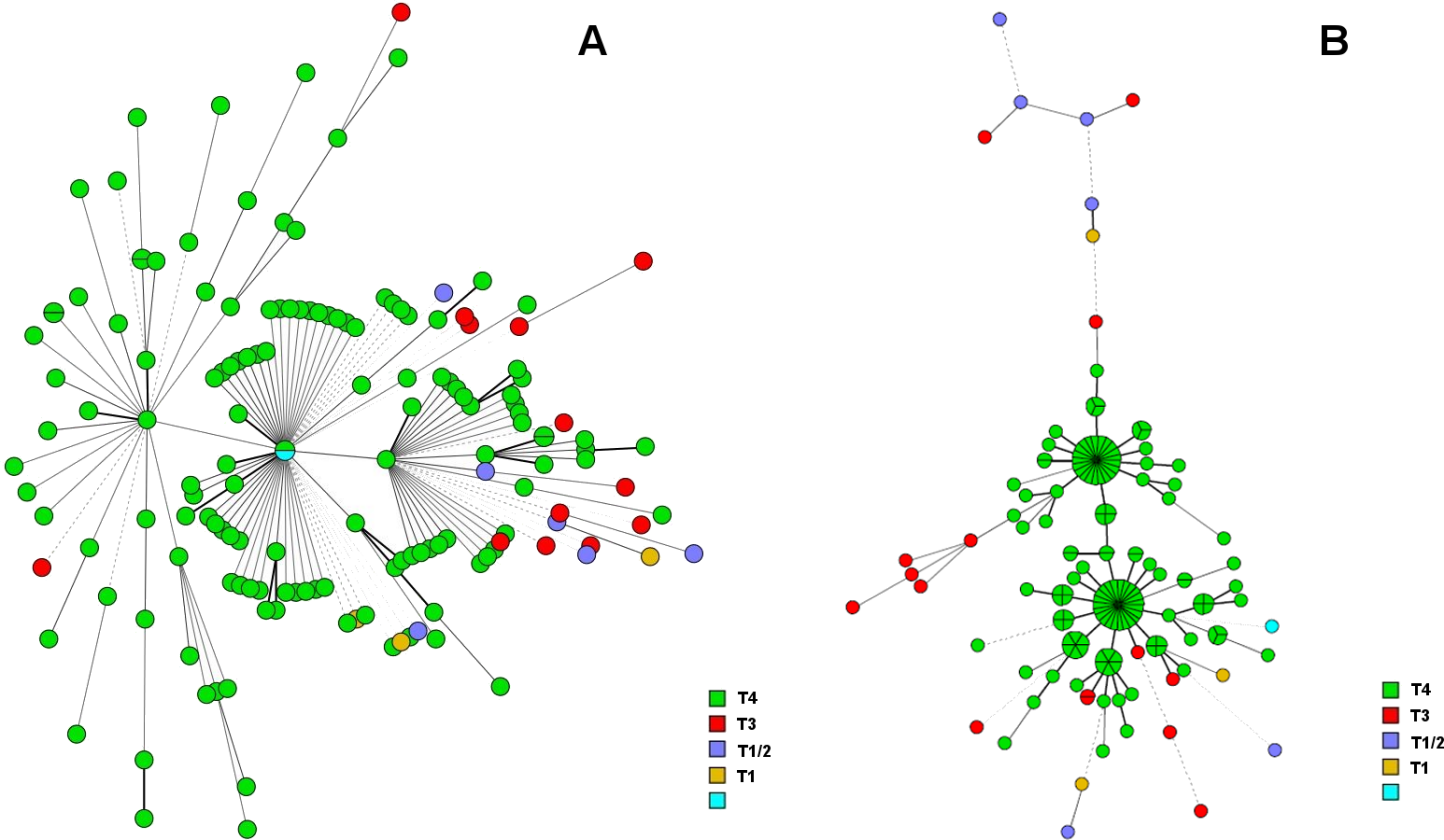
Two minimum spanning trees constructed on a UPGMA clustering on a similarity matrix that was calculated using categorical similarity index and allele copy numbers of all 17 microsatellites (A) and SNP-Type or leaving out the four with highest SI (B). The colors represent the SNP-Types as indicated in the indent; dark blue: no sequence available to differentiate type 1 and 2; light blue: no SNP type available. In Figure B, the node size represents the number of patients included.

When UPGMA based dendrograms including all 17 satellites and with or without including SNP-Type were constructed, most isolates belonged to two major groups. The isolates that were not of the SNP-Type 4 were observed at the outer limits of the tree (**S1 Fig).** For evaluation of the bacteriological population structure and the influence of inclusion of loci on cluster formation and tree topography, we constructed a MST including either all 17 satellites or gradually removing the most variable ones. As observed in organizing the allele number in an Excel file, the same three clusters of genotype pairs were observed in the MST when including all 17 loci, and the 20 clusters when omitting the four most variable markers (**Fig 3**). Depending on the number of VNTRs included for MST construction, we observed either two or three major groups and gradually excluding VNTRs with the highest variability, we observed that AC9 or/and AC8b are the main drivers for maintaining separate groups; omitting these markers resulted in a population with a large central cluster of 88 isolates with 11 branches. Most of the isolates have indeed a 6- or 7-copy number of these alleles and leaving out these markers coincides with the observation of clusters formed by different SNP-Types (**S5 Fig**).

## Discussion

In 1991, the WHO adopted a resolution for elimination of leprosy by the year 2000 and implementation of MDT resulted in a significant reduction of prevalence. Between 2002 and 2012, a 65% reduction in the prevalence (from 4.33 to 1.51 patients/10,000) was achieved in Brazil but leprosy is unevenly distributed within the country with pockets of incidence levels of more than 10/10,000 [44]. The Northeast region is the poorest of the country reporting a third of the newly diagnosed patients and a detection rate that is twice that of the average in the country and the State of Ceará is one of the poorest states in the region. Over 10% of its municipalities classified as hyperendemic and the capital, Fortaleza, considered a priority for leprosy control, having the highest demographic density in the country and one of the municipalities in the state with the highest detection rates [45]. In addition, 5.9% of the new patients are less than 15 years of age and only half of the contacts are being investigated for disease [33].

Transmission of leprosy is assumed to be from person to person through the respiratory system or damaged skin, with risk for developing disease being higher if a family member had disease and even more when these presenting the LL form [46, 47]. However, new patients often mention lack of contact with other leprosy patients, suggestive of unrecognized transmission routes [48], including exposure to an environmental source such as water, soil, plants and animals [49] but no study unequivocally demonstrated the mechanism of leprosy transmission [50].

Since the report on the existence of genetic variability [4, 5] and of the genome sequence of *M*. *leprae* [6], analysis of SNP-Types and micro- and mini-satellites added to our knowledge about genetic variability of *M*. *leprae* and its biology, such as existence of geographic or family associated genotypes [18, 19, 23], genetic divergence between bacilli inhabiting different tissue [20] and differentiation between relapse and re-infection [14]. Although studies on genetic variability of *M*. *leprae* have been conducted in several regions endemic for leprosy, mostly detailed epidemiologic information is missing except for a study in Qiubei, China, demonstrating intra-familial strain types [19] and regional differences in clustering [18]. No prospective molecular epidemiology study with detailed epidemiologic and clinical data have been reported except for a study reporting transmission of dapsone resistant *M*. *leprae* in Cebu, the Phillipines [51].

We hereby confirm the high prevalence of SNP-Type 4 in the northeast of Brazil as reported previously [16] and probably due to introduction of leprosy by slave traffic from West Africa. Isolates with SNP-Type 3 are partly 3I, as defined by the *gyr*A97 SNP (SNP7614) [52]; and our earlier observation during studies on drug resistance [14, 53]. We also observed a surprising strong correlation between SNP- based and VNTR based genotypes suggesting that in certain populations, microsatellites are also deeply rooted into the bacterial population structure. Only by omitting GTA9 and AC8a from the analysis, the relation between VNTR and SNP-Type was disrupted. Association between certain VNTRs and SNP-Type has been demonstrated before [15, 31] but might be more pronounced here due to the very high level of SNP-Type 4 in our study population. Because of the high level of SNP-Type 4 in the studied population, it would have been interesting to characterize the *M*. *leprae* isolates to the sub-SNP-Type level but no DNA was left to perform this.

The influence of stringency of definition of genotype clustering for interpretation of transmission and phylogeny has been clearly demonstrated for tuberculosis [54] but not extensively for leprosy [26]. The difference in clustering level using two stringencies in the present study is remarkable (3.8% vs. 60.4%) and we believe that the high clustering level represents recent transmission and therefore being the major drive for developing leprosy in Fortaleza. Although clusters are generally small, we also observed two larger ones and clusters of considerable size have also been described in China [18], in the Philippines [26, 27] and among those shared between humans and armadillos in the US [32]. The choice of stringency for definition of clustering in the present study is partly based on the fact that the four markers with Simpson Index >0.85 also were mostly presenting allele differences in the genotypes of *M*. *leprae* present in the nose and in the skin. Those were also among those disfavoring MLVA analysis for *M*. *leprae* genotyping as described by Monot et al. [8]. One weakness of our study is that we have no epidemiologic links that proovef that the 13 VNTR-based clustering is indicative for intense leprosy transmission in the present setting but this is probably due to lack of healthy household contacts (HHC) in the present sampling and low representativity of sampling. Extensive MIRU-VNTR genotyping data from *M*. *tuberculosis* show that the most variable MIRUs can be omitted without much loss of transmission links [54]. The number of *M*. *leprae* bacilli in the human body can reach 10^12^ so differences in copy number due to higher number of replication cycles during development of leprosy are imaginable. Finally, the presently used VNTR-based stringency is still higher than that those used by Sharma et al. [32] and Lavania et al. [24]. Sharma et al. related the SNP-VNTR type 3I-2-v1 genotype among 80.3% of the armadillo samples from the South of the US and 22/52 human patients were infected with *M*. *leprae* presenting one of two major genotypes. Interestingly, Lavania et al. [24], using an identical typing approach observed 66 different patterns among 70 leprosy patients. Although sample representativity and other variables might strongly influence clustering levels, the difference between both studies is striking and might also be due to differences in transmission dynamics. Some markers that were included [(TA)10 and 18–8] were not used for genotype definition in the before mentioned studies but in the present study had a HGDI of 0.38 and 0.22, respectively. This again suggests the need for regional evaluation of VNTRs for local *M*. *leprae* genotyping for developing "lower cost" genotyping in the mostly poorer endemic regions. However, having in mind the huge amount of information obtained from the standardized 24-MIRU-VNTR procedures for phylogenetic studies of *M*. *tuberculosis*, we here suggest the use of 17 STRs or even more for better understanding of transmission and phylogeny of *M*. *leprae* on a larger scale.

The comparison of *M*. *leprae* genotypes present in skin biopsy and nasal secretion is described and discussed in detail elsewhere [43]. While all isolates presently presented four copies of (GGT)5, one nasal swab sample presented six copies of this allele [43] and although other alleles than that of four copies are described with very low frequency in Brazil [16], they are more frequent in countries like Thailand [27] and the Philippines [25]. Contrary to the single allele with two copies of 23–3 described by Lima et al. [43], in 8% of our patients, a single copy of this marker was observed. A further finding by Lima et al. was the observation that some individuals presented differences in copy number in five to seven loci, including less variable ones, being highly suggestive for multiple infection or more extensive intra-patient strain evolution. In addition and more importantly for transmission studies, our data show that inclusion of the genotypes from nasal swabs may have consequences for clustering outcome. Because the hypothesis is that the nose is a port of entry and exit of *M*. *leprae*, the genotype in nasal swabs could contribute to the transmission links suggested by genotyping *M*. *leprae* in skin biopsies. We therefore suggest that more studies including both samples are needed to understand transmission dynamics. However, as stated elsewhere, there is no guarantee that *M*. *leprae* in the nasal swab is representative for disease but very recently, molecular evidence for an important role of the nose in leprosy transmission was presented by Araujo et al. [55].

High levels of recent transmission in Fortaleza is also evidenced by the observation of two large clusters of about 20 patients and may indicate the existence of two main lineages of *M*. *leprae* strains differing in four alleles (AC8b, GTA9, AC9 and AC8a) in Fortaleza. This might be related to some undetected factor causing more transmission of these strains but unfortunately, our study did not allow their definition and might depend on a social network approach as demonstrated in molecular epidemiology studies of tuberculosis [56]. Alternatively, these strains might have higher transmissibility, undescribed so far in leprosy but proven for some lineages of *M*. *tuberculosis*. Our earlier observation that reinfection or strain selection of *M*. *leprae* isolates of SNP-Type 4 was very frequent in relapse patients in Rio de Janeiro, a region predominant for SNP-Type 3 could be an example of that [14].

Identifying behavioral and environmental risk factors for developing leprosy is a difficult task because of the long incubation time of the disease (2–5 years for tuberculoid leprosy and 8–12 years for lepromatous leprosy). It is not easy to determine time and duration of exposure and onset of infection and risk factors for disease might change over time. Among 165 municipalities in the state of Ceará, a 300-fold difference in disease incidence was observed and associated with poverty, inequality, uncontrolled urbanization, population growth and low level of education [57]. The same group [44] also looked for socioeconomic, environmental and behavioral factors associated with leprosy in a case control study in four municipalities including that of Fortaleza; low education level, experience of food shortage at any time in life, frequent contact with natural bodies of water and infrequent changing of bed linen were associated with leprosy. Another study in this city concentrated on infection with *M*. *leprae* in the absence of clinical disease and demonstrated that higher levels of anti PGL-1 in patients without known contact with leprosy patients are much higher than reported elsewhere in the literature [58]. More recently, nasal carriage of *M*. *leprae* by PCR was observed in 67% of HHC but interestingly, 28% of persons living in richer part of the city were also positive. This is probably due to complex interaction between the populations at high and low risk for infection by leprosy. Domestic service and daily migration of the poor in houses of the upper class and richer parts of the city is still common [41].

An earlier spatial analysis in Ceará showed the highest density of disease is among the most urbanized and economically highest developed [59]. Our spatial analysis on genotype distribution did not demonstrate a distribution of clustering that was different from disease distribution in Fortaleza in general, showing that with the present data, there do not seem to be clear hot spots of (recent) transmission in the city. However, some neighborhoods were associated with the two biggest clusters, being group 12 (Jacareacanga, Canindezinho, Conjunto Esperança and Manoel Sátiro) and group 14 (Bonsucesso and Vila Pery). We also observed that three of these neighborhoods (Bonsucesso, Canindezinho and Conjunto Esperança) presented patients with high BI (note that only MB cases were submitted to genotyping) and in a recent study on the social, educational and economic development of neighborhoods in Fortaleza, both were indicated as being among the poorest in the city (www.ipece.ce.gov.br/publicacoes/Perfil%20Socioeconomico%20Fortaleza%20final-email.pdf). In addition, very recent data also demonstrate that both neighborhoods are hyperendemic (> 4/10.000) for leprosy with high incidence in children less than 15 years of age (0.5-1/10.000) [60].

Some limitations of our study is that our sampling occurred during a relatively short period of time, that genotyping was performed only on 15% of the new MB patients and that PB patients were omitted from analysis. This might mask transmission links due to factors other than contact with MB patients and explain why a considerable proportion of the new patients were not aware of earlier contact with patients. Nonetheless, the most significant association with clustering was having positive bacilloscopy, which is in agreement with the long standing idea that transmission of leprosy is caused by close contact with MB patients. However, significance of this finding is weakened because the mean BI between groups with clustered and unique genotypes is almost the same, but again, only MB patients were submitted to genotyping. Definition of being MB or PB in the present study is based on Ridley-Jopling method and our results are in favor for maintaining this technique as part of the diagnostic procedure, contrary to the current recommendation of WHO to define PB and MB patients only on basis of number of lesions and nerve involvement.

The significant association of clustering with patients having had contact with another case at work but not at time of diagnosis present could be due to the long incubation time for developing leprosy; however, a low number of patients reported contact at work. Although we could not establish a relation of cluster with the nature of the work or localization of the workplace, this needs further investigation because some working places harbor a large number of persons including undetected leprosy cases during long periods and could be hot spots of transmission. Some examples are metallurgic and car assembly factories, areas of civil construction, handicraft fairs and offices. Social interactions and the physical, residential and occupational environments have been suggested to be more conducive to transmission of a community in Qiubei, China [18]. This finding is not in line with our observation that having lived with a leprosy patient is associated with belonging to a non-cluster and to explain this, further research, eventually using whole genome sequencing is warrented.

HHC have been described to be at higher risk for developing leprosy in several conventional epidemiologic studies but also in studies that performed *M*. *leprae* genotyping, including China [17], Thailand [27], Colombia [31] and India [24]. Although investigation of HHC is part of the leprosy program in Brazil, this is not always being performed and in Fortaleza in particular, this seems to be the case in about 50% of the patients [43]. The lack of association between clustering and house hold in the present study is probably due to the inclusion of new patients only and without contact investigation and inclusion of patients from the same house hold. Nonetheless, our observation of inversed association of sharing home with a leprosy case and cluster is surprising and needs to be better investigated.

Another puzzling finding was the significant association between alcohol use and having *M*. *leprae* with a unique genotype. Several studies associated alcohol (ab)use as a risk factor for leprosy, including a case control study in Mato Grosso state [61], Maranhao state [62] and with treatment abandonment in Tocantins [63]. This finding needs further investigation but again, the low number of patients in some analytical cells due to the paucity of biopsied patients and lack of specific questionnaire data could be partly responsible. Another issue are the different protocols used for collecting information about alcohol (ab)use.

We also observed that some characteristics that are usually associated with higher risk for leprosy also had a tendency to be more pronounced in clustered patients. This was the case of clustering among males and later diagnosis at a later stage due to more reluctance to seek care among men as widely in Brazil. We also observed a tendency to have a higher disability grade in clustered patients. Higher disability grade reflects longer incubation time, bacillary load and time before diagnosis, therefore being able to infect more individuals. This is in concordance with the longer time delay between first observation of lesions and disease diagnosis reported in clustered patients.

We conclude by referring to a very recent study that evaluated temporal trends in leprosy in Fortaleza for the period 2001 to 2012 [59]. Although there was a steady decrease in the number of new patients, from hyperendemic (≥4/10,000) in 2001 to highly endemic (2<4/10,000) in 2012, the number of new patients in children less than 15 years old was steady and there was also noted a steady increase in the number of MB and of lepromatous patients since 2005. Such data indicate both ongoing recent transmission including to children and late diagnosis in adults, reflected also by the rise in grade 2 disability (from 6% to 9% in new patients). Given the chronic nature and natural history of the disease it is unlikely that there will be an improvement of these trends in the near future. Low levels of education, unfavorable socioeconomic conditions, and delayed presentation to the health system are factors that are generally associated with late diagnosis. This is in agreement with our data of high clustering levels and, demonstrating that recent transmission of leprosy is a serious problem in Fortaleza. The realization of a prospective molecular epidemiologic study in a complex setting like Fortaleza is difficult but we hope that a new study of longer duration, with higher intake of patients, collecting both skin biopsy and nasal swabs or biopsy, inclusion of HHC, a more detailed questionnaire including social network studies that might allow definition of risk factors for belonging to the same cluster, and finally investment in DNA extraction and more sensitive genotyping that allows inclusion of PB patients. As a final comment, we believe that, although whole genome sequencing of *M*. *leprae* genomes is still challenging because of the need of bacterial DNA enrichment, the technical expertise needed and the considerable cost, inclusion in future studies might be beneficial for better understanding of leprosy transmission.

## Supporting information

S1 Fig A UPGMA based dendrogram was constructed based on a similarity matrix constructed on the categorical values from 17 micro- and minisattelites from 159 isolates of *M*. *leprae*. The SNP types were not included for calculation of the similarity matrix.
(TIF)

S2 Fig Number of leprosy cases as recorded during the time period of study. Time of diagnosis was available for 154 patients and 92 of whom had *Mycobacterium leprae* presenting a clustered genotype, including 23 and 19 patients belonging respectively to cluster 14 and cluster 12.
(TIF)

S3 Fig The figure represents the different neighborhoods and data on the population in Fortaleza in 2010, on leprosy cases in the same period and on 156 cases diagnosed in 2009 and 2010 with genotyped *M*. *leprae*. The panels show the spatial distribution in the city of Fortaleza (A), the number of cases per neighborhood (B), their estimated population (C), application of the Kernel density estimation on the leprosy cases with genotypes (D) and using simple (E) or dual (F) Kernel density estimation. The neighborhoods indicated by arrows indicate higher density.
(TIF)

S4 Fig Geographic distribution of the 156 genotyped cases indicating their number of lesions (A) and their bacillary load, BAAR (C). For evaluation of eventual geographic concentration of cases with high level characteristics, we used inverse distance weighting (IDW) (B and D).
(TIF)

S5 Fig Three MST were built based on similarity matrices build comparing categorical values of the MLVA based alleles but cumulative omission of those with the highest discriminatory index, being (AT)15, (TA)18 and (AT)17 (A), minus (GAA)21 and (GTA)8 (B) and minus (AC)8a, (AC)9 and (AC)8b (C). The colors represent the SNP types as indicated in the indent; dark blue: no sequence available to differentiate type 1 and 2; light blue: no SNP type available. In Figure B, node size represents number of cases included.
(TIF)

S1 Table Genotypes based on MLVA- and SNP based analysis in skin biopsies samples. (DOC)

S2 Table Bivariate analysis of demographic, socioeconomic, behavioral, and environmental variables with leprosy. (DOC)

S3 Table Increase in clustering by inclusion of genotypes of *Mycobacterium leprae* present in nasal swabs. (DOC)

S4 Table Distribution of genotyped cases among the neighborhoods of Fortaleza. (DOC)
