# Supplementary figures and images for: Genotyping of Mycobacterium leprae for better understanding of leprosy transmission in Fortaleza, Northeastern Brazil
Source: PLoS Negl Trop Dis. 2017 Dec 15;11(12):e0006117. doi: 10.1371/journal.pntd.0006117 (PMC5747459; doi:10.1371/journal.pntd.0006117)

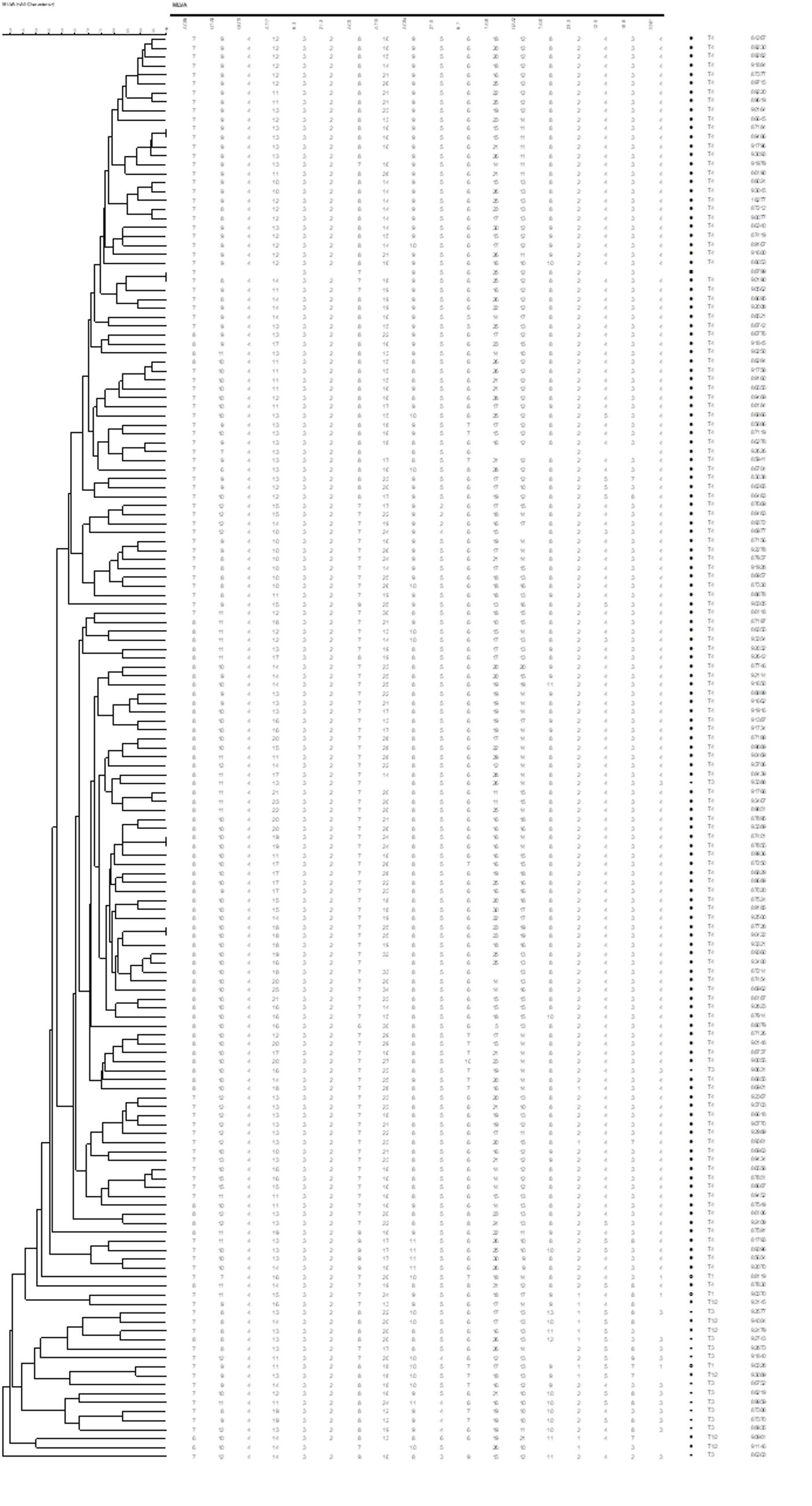

Supplement: S1 Fig — The SNP types were not included for calculation of the similarity matrix. (TIF) [file pntd.0006117.s001.tif]

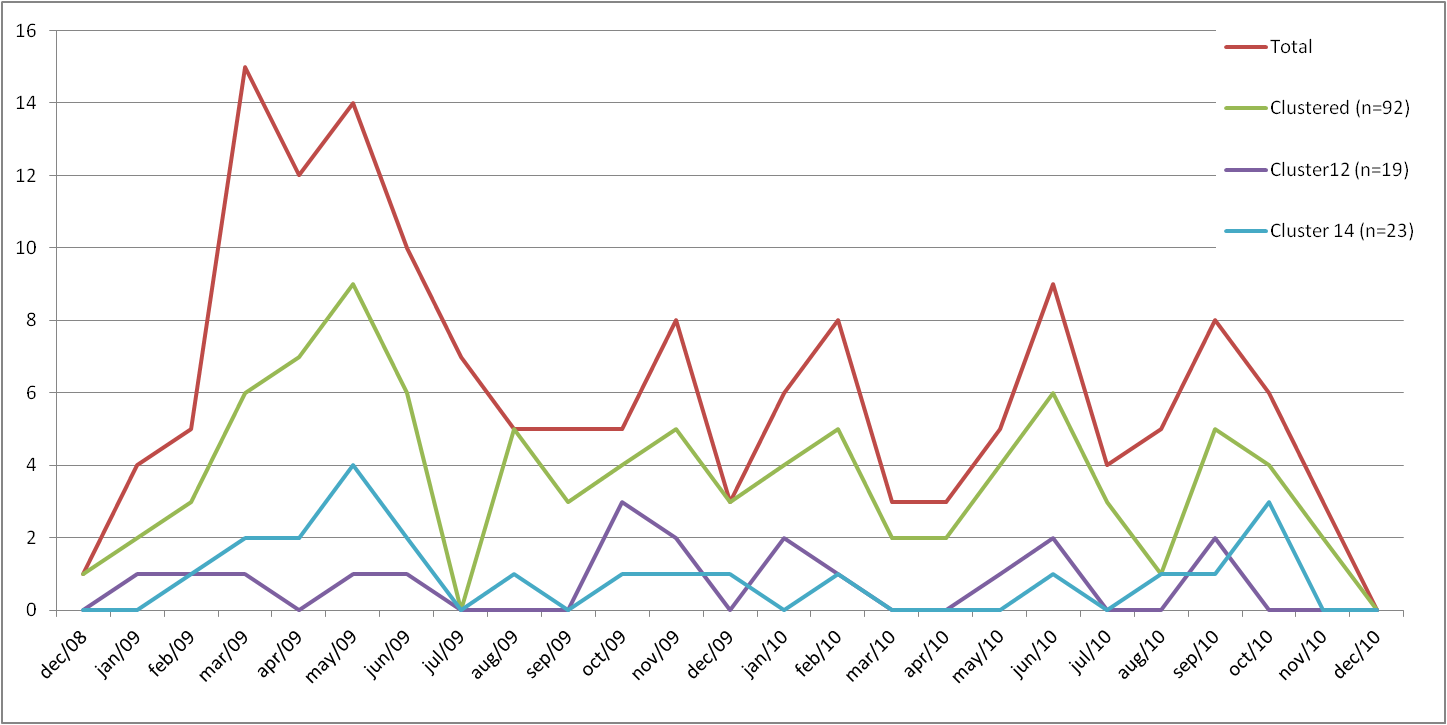

Supplement: S2 Fig — Time of diagnosis was available for 154 patients and 92 of whom had Mycobacterium leprae presenting a clustered genotype, including 23 and 19 patients belonging respectively to cluster 14 and cluster 12. (TIF) [file pntd.0006117.s002.tif]

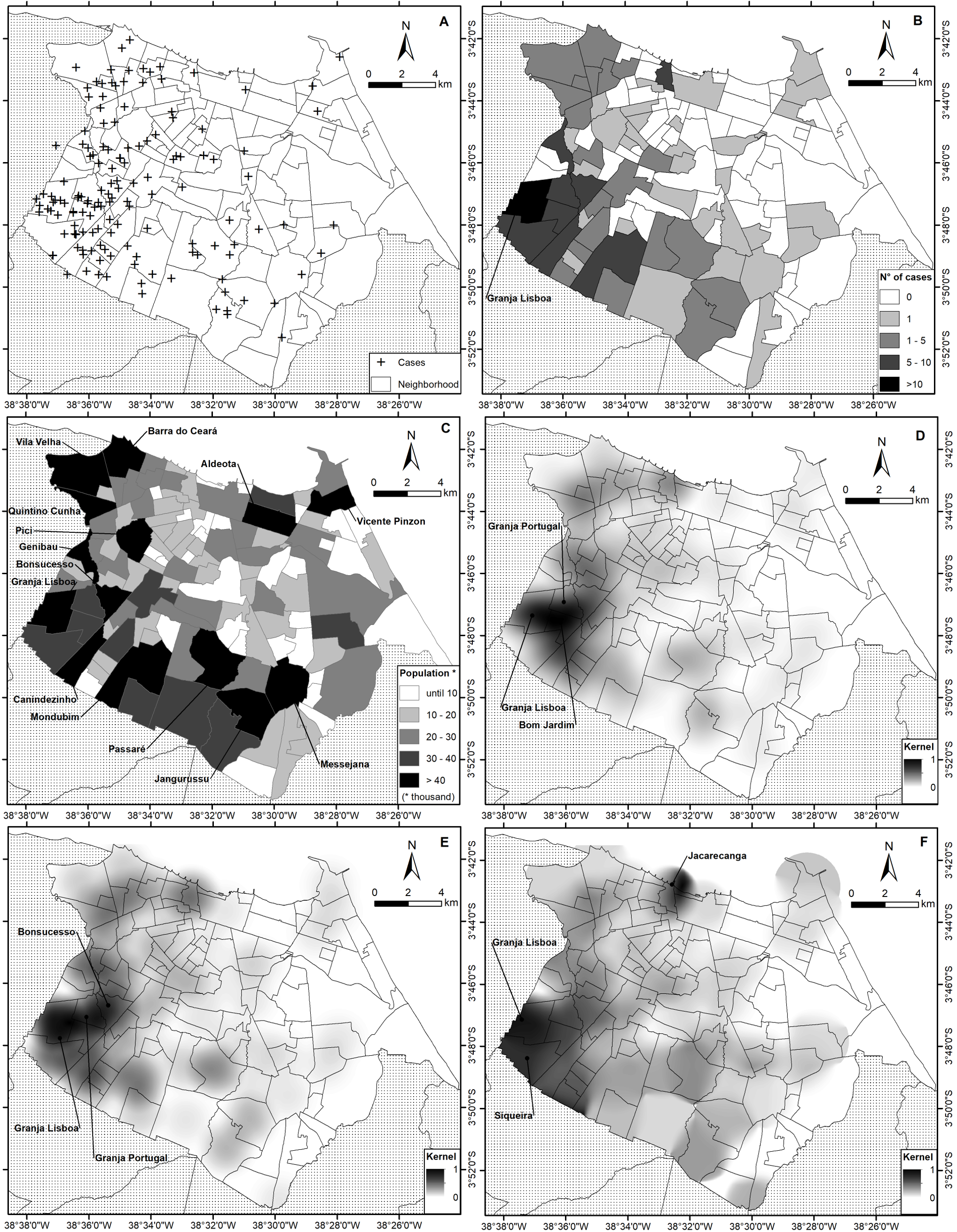

Supplement: S3 Fig — The panels show the spatial distribution in the city of Fortaleza (A), the number of cases per neighborhood (B), their estimated population (C), application of the Kernel density estimation on the leprosy cases with genotypes (D) and using simple (E) or dual (F) Kernel density estimation. The neighborhoods indicated by arrows indicate higher density. (TIF) [file pntd.0006117.s003.tif]

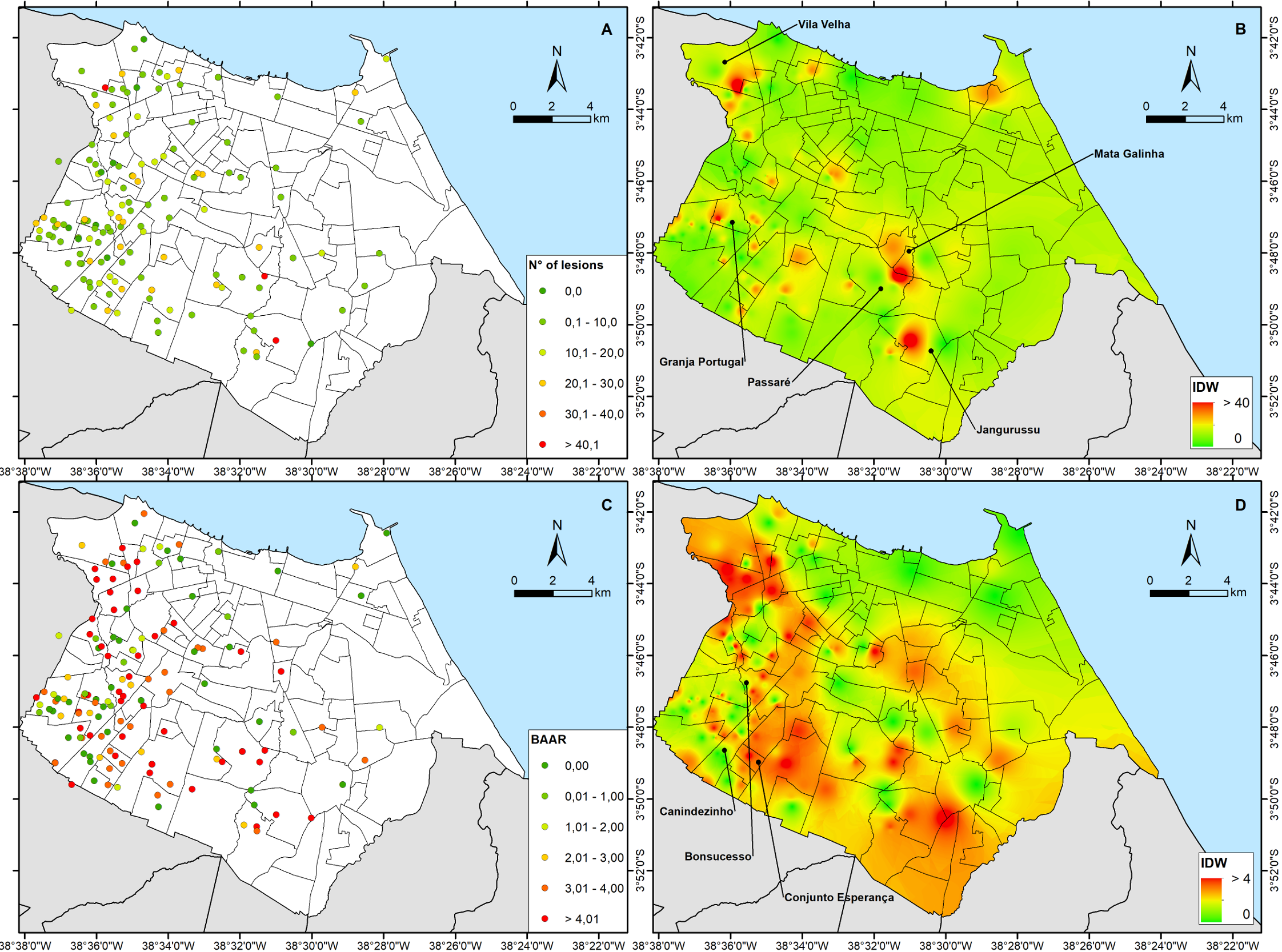

Supplement: S4 Fig — Geographic distribution of the 156 genotyped cases indicating their number of lesions (A) and their bacillary load, BAAR (C). For evaluation of eventual geographic concentration of cases with high level characteristics, we used inverse distance weighting (IDW) (B and D). (TIF) [file pntd.0006117.s004.tif]

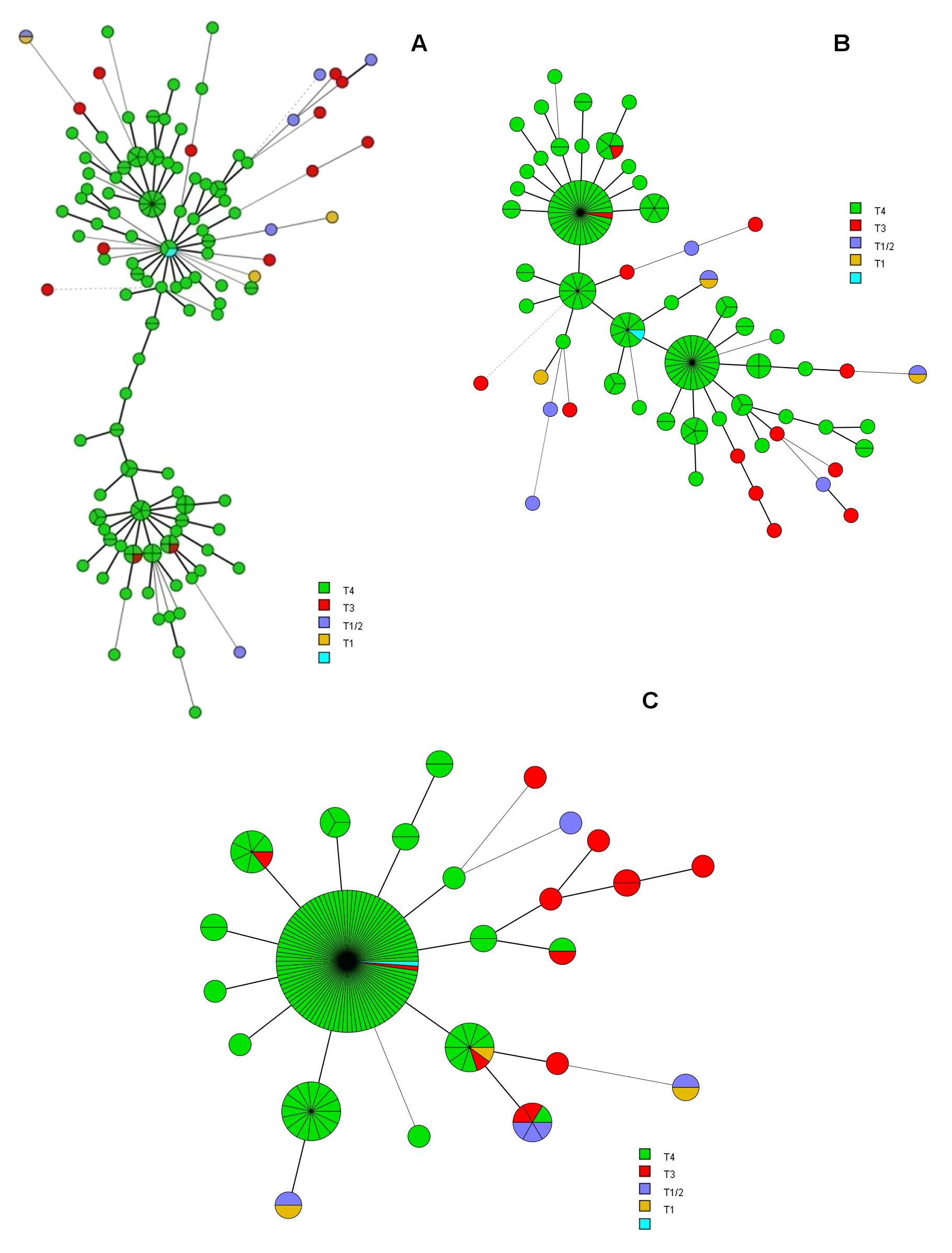

Supplement: S5 Fig — Three MST were built based on similarity matrices build comparing categorical values of the MLVA based alleles but cumulative omission of those with the highest discriminatory index, being (AT)15, (TA)18 and (AT)17 (A), minus (GAA)21 and (GTA)8 (B) and minus (AC)8a, (AC)9 and (AC)8b (C). The colors represent the SNP types as indicated in the indent; dark blue: no sequence available to differentiate type 1 and 2; light blue: no SNP type available. In Figure B, node size represents number of cases included. (TIF) [file pntd.0006117.s005.tif]
