# Supplementary material for: Genotyping of Mycobacterium leprae for better understanding of leprosy transmission in Fortaleza, Northeastern Brazil
Source: PLoS Negl Trop Dis. 2017 Dec 15;11(12):e0006117. doi: 10.1371/journal.pntd.0006117 (PMC5747459; doi:10.1371/journal.pntd.0006117)
Supplement: S1 Table — (DOC) [file pntd.0006117.s006.doc]

**S1 Table.** Genotypes based on MLVA- and SNP based analysis in skin biopsies samples.

| **Sample** | **Genetic marker** | | | | | | | | | | | | | | | | | |
| --- | --- | --- | --- | --- | --- | --- | --- | --- | --- | --- | --- | --- | --- | --- | --- | --- | --- | --- |
|  | **AC8b** | **GTA9** | **GGT5** | **AT17** | **6-3** | **21-3** | **AC9** | **AT15** | **AC8a** | **27-5** | **6-7** | **TA18** | **GAA21** | **TA10** | **23-3** | **12-5** | **18-8** | **SNP** |
| **18277** | 7 | 9 | 4 | 12 | 3 | 2 | 8 | 14 | 9 | 5 | 6 | 25 | 13 | 8 | 2 | 4 | 3 | 4 |
| **81793** | 7 | 11 | 4 | 13 | 3 | 2 | 9 | 17 | 11 | 5 | 6 | 26 | 10 | 8 | 2 | 5 | 8 | 4 |
| **83038** | 7 | 9 | 4 | 13 | 3 | 2 | 8 | 23 | 9 | 5 | 6 | 17 | 12 | 8 | 2 | 5 | 7 | 4 |
| **84267** | 7 | 9 | 4 | 12 | 3 | 2 | 8 | 16 | 9 | 5 | 6 | 18 | 12 | 8 | 2 | 4 | 3 | 4 |
| **85654** | 7 | 10 | 4 | 13 | 3 | 2 | 9 | 17 | 11 | 5 | 6 | 30 | 9 | 8 | 2 | 4 | 3 | 4 |
| **85886** | 7 | 9 | 4 | 13 | 3 | 2 | 8 | 18 | 9 | 5 | 7 | 17 | 12 | 8 | 2 | 4 | 3 | 4 |
| **85941** | 7 | 9 | 4 | 13 | 3 | 2 | 8 | 17 | 8 | 5 | 7 | 31 | 12 | 8 | 2 | 4 | 3 | 4 |
| **86106** | 8 | 12 | 4 | 13 | 3 | 2 | 7 | 20 | 8 | 5 | 8 | 23 | 13 | 8 | 2 | 4 | 3 | 4 |
| **86118** | 7 | 11 | 4 | 12 | 3 | 2 | 7 | 30 | 8 | 5 | 6 | 18 | 15 | 8 | 2 | 4 | 3 | 4 |
| **86187** | 8 | 10 | 4 | 21 | 3 | 2 | 7 | 23 | 8 | 5 | 6 | 15 | 15 | 8 | 2 | 4 | 3 | 4 |
| **86190** | 7 | 9 | 4 | 11 | 3 | 2 | 8 | 28 | 9 | 5 | 6 | 21 | 11 | 8 | 2 | 4 | 3 | 4 |
| **86278** | 7 | 9 | 4 | 13 | 3 | 2 | 8 | 18 | 8 | 5 | 6 | 16 | 12 | 8 | 2 | 4 | 3 | 4 |
| **86303** | 7 | 12 | 4 | 14 | 3 | 2 | 9 | 16 | 8 | 3 | 9 | 15 | 12 | 11 | 2 | 4 | 2 | 3 |
| **86340** | 7 | 9 | 4 | 13 | 3 | 2 | 8 | 14 | 9 | 5 | 6 | 30 | 12 | 9 | 2 | 4 | 3 | 4 |
| **86483** | 7 | 10 | 4 | 12 | 3 | 2 | 8 | 17 | 9 | 5 | 6 | 19 | 12 | 8 | 2 | 5 | 8 | 4 |
| **86555** | 7 | 10 | 4 | 11 | 3 | 2 | 8 | 16 | 9 | 5 | 6 | 21 | 12 | 8 | 2 | 4 | 3 | 4 |
| **86558** | 7 | 10 | 4 | 16 | 3 | 2 | 7 | 16 | 8 | 5 | 6 | 14 | 12 | 8 | 2 | 4 | 3 | 4 |
| **86618** | 7 | 12 | 4 | 13 | 3 | 2 | 7 | 18 | 8 | 5 | 6 | 19 | 13 | 8 | 2 | 4 | 3 | 4 |
| **86645** | 7 | 9 | 4 | 12 | 3 | 2 | 8 | 13 | 9 | 5 | 6 | 23 | 14 | 8 | 2 | 4 | 3 | 4 |
| **86704** | 7 | 6 | 4 | 13 | 3 | 2 | 8 | 16 | 10 | 5 | 8 | 28 | 12 | 8 | 2 | 4 | 3 | 4 |
| **86752** | 7 | 9 | 4 | 14 | 3 | 2 | 8 | 18 | 10 | 5 | 7 | 16 | 12 | 9 | 2 | 4 | 3 | 3 |
| **86776** | 8 | 9 | 4 | 13 | 3 | 2 | 8 | 22 | 9 | 5 | 6 | 17 | 12 | 8 | 2 | 4 | 3 | 4 |
| **86829** | 8 | 10 | 4 | 17 | 3 | 2 | 7 | 28 | 8 | 5 | 6 | 19 | 18 | 8 | 2 | 4 | 3 | 4 |
| **86855** | 8 | 10 | 4 | 14 | 3 | 2 | 7 | 25 | 9 | 5 | 7 | 20 | 14 | 8 | 2 | 4 | 3 | 4 |
| **86866** | 7 | 10 | 4 | 13 | 3 | 2 | 8 | 15 | 10 | 5 | 6 | 25 | 12 | 8 | 2 | 5 | 3 | 4 |
| **86901** | 8 | 10 | 4 | 18 | 3 | 2 | 7 | 28 | 8 | 5 | 7 | 16 | 14 | 8 | 1 | 4 | 3 | 4 |
| **86957** | 7 | 8 | 4 | 10 | 3 | 2 | 7 | 25 | 9 | 5 | 6 | 18 | 13 | 8 | 2 | 4 | 3 | 4 |
| **86962** | 8 | 10 | 4 | 25 | 3 | 2 | 7 | 34 | 8 | 5 | 6 | 14 | 16 | 8 | 2 | 4 | 3 | 4 |
| **86963** | 7 | 10 | 4 | 10 | 3 | 2 | 7 | 21 | 8 | 5 | 6 | 16 | 12 | 9 | 2 | 4 | 3 | 4 |
| **86977** | 7 | 12 | 4 | 10 | 3 | 2 | 7 | 24 | 9 | 4 | 6 | 15 | ND | 8 | 2 | 3 | 3 | 4 |
| **87020** | 8 | 9 | 4 | 17 | 3 | 2 | 7 | 23 | 8 | 5 | 6 | 16 | 16 | 8 | 2 | 4 | 3 | 4 |
| **87119** | 7 | 10 | 4 | 13 | 3 | 2 | 8 | 16 | 9 | 5 | 7 | 15 | 12 | 8 | 2 | 4 | 3 | 4 |
| **87126** | 8 | 10 | 4 | 12 | 3 | 2 | 7 | 29 | 8 | 5 | 7 | 17 | 14 | 8 | 2 | 4 | 3 | 4 |
| **87156** | 7 | 9 | 4 | 10 | 3 | 2 | 7 | 16 | 9 | 5 | 6 | 19 | 14 | 8 | 2 | 4 | 3 | 4 |
| **87184** | 7 | 9 | 4 | 13 | 3 | 2 | 8 | 16 | 9 | 5 | 6 | 15 | 11 | 8 | 2 | 4 | 3 | 4 |
| **87188** | 8 | 10 | 4 | 20 | 3 | 2 | 7 | 28 | 8 | 5 | 6 | 17 | 14 | 8 | 2 | 4 | 3 | 4 |
| **87197** | 8 | 11 | 4 | 18 | 3 | 2 | 7 | 21 | 9 | 5 | 6 | 10 | 15 | 8 | 2 | 4 | 3 | 4 |
| **87212** | 7 | 8 | 4 | 12 | 3 | 2 | 8 | 14 | 9 | 5 | 6 | 23 | 13 | 8 | 2 | 4 | 3 | 4 |
| **87214** | 8 | 10 | 4 | 18 | 3 | 2 | 7 | 33 | 8 | 5 | 6 | 23/25 | 13 | 8 | 2 | 4 | 3 | 4 |
| **87250** | 8 | 10 | 4 | 17 | 3 | 2 | 7 | 26 | 8 | 5 | 7 | 16 | 15 | 8 | 2 | 4 | 3 | 4 |
| **87306** | 7 | 8 | 4 | 19 | 3 | 2 | 8 | 12 | 9 | 4 | 7 | 19 | 10 | 10 | 2 | 4 | 3 | 3 |
| **87330** | 7 | 8 | 4 | 10 | 3 | 2 | 7 | 26 | 10 | 5 | 6 | 18 | 16 | 8 | 2 | 4 | 3 | 4 |
| **87370** | 7 | 9 | 4 | 19 | 3 | 2 | 8 | 12 | 9 | 4 | 7 | 19 | 10 | 10 | 2 | 5 | 8 | 3 |
| **87377** | 7 | 9 | 4 | 12 | 3 | 2 | 8 | 21 | 9 | 5 | 6 | 16 | 12 | 8 | 2 | 4 | 3 | 4 |
| **87401** | 8 | 10 | 4 | 19 | 3 | 2 | 7 | 24 | 8 | 5 | 6 | 16 | 14 | 8 | 2 | 4 | 3 | 4 |
| **87419** | 7 | 9 | 4 | 12 | 3 | 2 | 8 | 15 | 9 | 5 | 6 | 15 | 12 | 9 | 2 | 4 | 3 | 4 |
| **87454** | 8 | 10 | 4 | 20 | 3 | 2 | 7 | 20 | 8 | 5 | 6 | 14 | 13 | 8 | 2 | 4 | 3 | 4 |
| **87524** | 8 | 10 | 4 | 15 | 3 | 2 | 7 | 18 | 8 | 5 | 6 | 20 | 18 | 8 | 2 | 4 | 3 | 4 |
| **87549** | 8 | 10 | 4 | 11 | 3 | 2 | 7 | 16 | 9 | 5 | 6 | 14 | 13 | 8 | 2 | 4 | 3 | 4 |
| **87591** | 8 | 11 | 4 | 19 | 3 | 2 | 9 | 16 | 9 | 5 | 6 | 22 | 11 | 9 | 2 | 4 | 3 | 4 |
| **87669** | 7 | 12 | 4 | 15 | 3 | 2 | 7 | 17 | 9 | 2 | 6 | 17 | 15 | 8 | 2 | 4 | 3 | 4 |
| **87728** | 8 | 10 | 4 | 18 | 3 | 2 | 7 | 25 | 8 | 5 | 6 | 23 | 19 | 8 | 2 | 4 | 3 | 4 |
| **87746** | 8 | 10 | 4 | 14 | 3 | 2 | 7 | 23 | 8 | 5 | 6 | 20 | 20 | 9 | 2 | 4 | 3 | 4 |
| **87830** | 8 | 11 | 4 | 14 | 3 | 2 | 7 | 19 | 8 | 5 | 8 | 31 | 12 | 8 | 2 | 5 | 8 | 4 |
| **87851** | 7 | 15 | 4 | 16 | 3 | 2 | 7 | 16 | 8 | 5 | 6 | 14 | 12 | 8 | 2 | 4 | 3 | 4 |
| **87855** | 8 | 10 | 4 | 19 | 3 | 2 | 7 | 24 | 8 | 5 | 6 | 16 | 14 | 8 | 2 | 4 | 3 | 4 |
| **87895** | 8 | 10 | 4 | 20 | 3 | 2 | 7 | 21 | 8 | 5 | 6 | 16 | 18 | 8 | 2 | 4 | 3 | 4 |
| **87914** | 8 | 10 | 4 | 16 | 3 | 2 | 7 | 15 | 8 | 5 | 6 | 18 | 15 | 10 | 2 | 4 | 3 | 4 |
| **87937** | 7 | 8 | 4 | 10 | 3 | 2 | 7 | 24 | 9 | 5 | 6 | 21 | 14 | 8 | 2 | 4 | 3 | 4 |
| **88024** | 7 | 9 | 4 | 10 | 3 | 2 | 8 | 14 | 9 | 5 | 6 | 15 | 13 | 8 | 2 | 4 | 3 | 4 |
| **88053** | 7 | 9 | 4 | 12 | 3 | 2 | 8 | 16 | 9 | 5 | 6 | 16 | 10 | 10 | 2 | 4 | 3 | 4 |
| **88079** | 8 | 10 | 4 | 16 | 3 | 2 | 6 | 30 | 8 | 5 | 6 | 5 | 13 | 8 | 2 | 4 | 3 | 4 |
| **88119** | 7 | 7 | 4 | 16 | 3 | 2 | 7 | 20 | 10 | 5 | 7 | 18 | 14 | 8 | 2 | 4 | 3 | 1 |
| **88184** | 7 | 10 | 4 | 11 | 3 | 2 | 8 | 17 | 9 | 5 | 6 | 17 | 12 | 9 | 2 | 4 | 3 | 4 |
| **88219** | 7 | 10 | 4 | 12 | 3 | 2 | 8 | 16 | 9 | 5 | 6 | 21 | 10 | 10 | 2 | 5 | 8 | 3 |
| **88284** | 8 | 10 | 4 | 11 | 3 | 2 | 8 | 15 | 8 | 5 | 6 | 26 | 12 | 8 | 2 | 4 | 3 | 4 |
| **88355** | 8 | 11 | 4 | 12 | 3 | 2 | 7 | 13 | 10 | 5 | 6 | 15 | 14 | 8 | 2 | 4 | 3 | 4 |
| **88365** | 7 | 9 | 4 | 12 | 3 | 2 | 8 | 20 | 9 | 5 | 6 | 17 | 10 | 8 | 2 | 5 | 3 | 4 |
| **88439** | 8 | 11 | 4 | 17 | 3 | 2 | 7 | 14 | 8 | 5 | 6 | 28 | 14 | 8 | 2 | 4 | 3 | 4 |
| **88483** | 7 | 12 | 4 | 15 | 3 | 2 | 7 | 22 | 9 | 2 | 6 | 18 | 14 | 8 | 2 | 4 | 3 | 4 |
| **88521** | 7 | 9 | 4 | 14 | 3 | 2 | 8 | 16 | 9 | 5 | 5 | 14 | 17 | 8 | 2 | 4 | 3 | 4 |
| **88687** | 7 | 15 | 4 | 15 | 3 | 2 | 7 | 16 | 8 | 5 | 6 | 14 | 12 | 8 | 2 | 4 | 3 | 4 |
| **88689** | 8 | 10 | 4 | 17 | 3 | 2 | 7 | 22 | 8 | 5 | 6 | 25 | 16 | 8 | 2 | 4 | 3 | 4 |
| **88695** | 7 | 8 | 4 | 14 | 3 | 2 | 8 | 19 | 9 | 5 | 6 | 26 | 12 | 8 | 2 | 4 | 3 | 4 |
| **88737** | 8 | 10 | 4 | 17 | 3 | 2 | 7 | 16 | 8 | 5 | 7 | 21 | 14 | 8 | 2 | 4 | 3 | 4 |
| **88742** | 7 | 9 | 4 | 13 | 3 | 2 | 8 | 15 | 9 | 5 | 5 | 25 | 13 | 8 | 2 | 4 | 3 | 4 |
| **88799** | 7 | ND | ND | ND | 3 | ND | 7 | ND | 9 | 5 | 6 | 25 | 12 | 8 | 2 | ND | 3 | ND |
| **88878** | 7 | 8 | 4 | 11 | 3 | 2 | 7 | 19 | 9 | 5 | 6 | 18 | 13 | 9 | 2 | 4 | 3 | 4 |
| **88899** | 8 | 9 | 4 | 13 | 3 | 2 | 7 | 22 | 8 | 5 | 6 | 19 | 14 | 9 | 2 | 4 | 3 | 4 |
| **88935** | 7 | 12 | 4 | 13 | 3 | 2 | 8 | 19 | 9 | 4 | 6 | 19 | 11 | 10 | 2 | 4 | 8 | 3 |
| **89160** | 7 | 10 | 4 | 11 | 3 | 2 | 8 | 15 | 8 | 5 | 6 | 21 | 12 | 8 | 2 | 4 | 3 | 4 |
| **89167** | 7 | 9 | 4 | 12 | 3 | 2 | 8 | 14 | 10 | 5 | 6 | 17 | 12 | 9 | 2 | 4 | 3 | 4 |
| **89185** | 8 | 10 | 4 | 15 | 3 | 2 | 7 | 18 | 8 | 5 | 6 | 30 | 17 | 8 | 2 | 4 | 3 | 4 |
| **89220** | 7 | 9 | 4 | 11 | 3 | 2 | 8 | 21 | 9 | 5 | 6 | 22 | 12 | 8 | 2 | 4 | 3 | 4 |
| **89230** | 7 | 9 | 4 | 12 | 3 | 2 | 8 | 16 | 9 | 5 | 6 | 20 | 12 | 8 | 2 | 4 | 3 | 4 |
| **89282** | 7 | 9 | 4 | 12 | 3 | 2 | 8 | 15 | 9 | 5 | 6 | 20 | 12 | 8 | 2 | 4 | 3 | 4 |
| **89360** | 8 | 10 | 4 | 19 | 3 | 2 | 7 | 32 | 8 | 5 | 6 | 25 | 13 | 8 | 2 | 4 | 3 | 4 |
| **89361** | 7 | 12 | 4 | 13 | 3 | 2 | 7 | 23 | 8 | 5 | 6 | 20 | 15 | 8 | 1 | 4 | 7 | 4 |
| **89372** | 7 | 12 | 4 | 14 | 3 | 2 | 7 | 19 | 9 | 2 | 6 | 16 | 17 | 8 | 2 | 4 | 3 | 4 |
| **89396** | 7 | 10 | 4 | 13 | 3 | 2 | 9 | 17 | 11 | 5 | 6 | 25 | 10 | 10 | 2 | 5 | 3 | 4 |
| **89434** | 7 | 13 | 4 | 13 | 3 | 2 | 7 | 23 | 8 | 5 | 6 | 21 | 12 | 9 | 2 | 4 | 3 | 4 |
| **89452** | 7 | 11 | 4 | 11 | 3 | 2 | 7 | 16 | 8 | 5 | 6 | 15 | 13 | 8 | 2 | 4 | 3 | 4 |
| **89469** | 7 | 10 | 4 | 12 | 3 | 2 | 8 | 16 | 8 | 5 | 6 | 28 | 12 | 8 | 2 | 4 | 3 | 4 |
| **89486** | 7 | 9 | 4 | 13 | 3 | 2 | 8 | 16 | 9 | 5 | 6 | 15 | 11 | 8 | 2 | 4 | 3 | 4 |
| **89619** | 7 | 9 | 4 | 11 | 3 | 2 | 8 | 21 | 9 | 5 | 6 | 25 | 12 | 8 | 2 | 4 | 3 | 4 |
| **89715** | 7 | 9 | 4 | 12 | 3 | 2 | 8 | 26 | 9 | 5 | 6 | 25 | 12 | 8 | 2 | 4 | 3 | 4 |
| **89851** | 8 | 11 | 4 | 22 | 3 | 2 | 7 | 20 | 8 | 5 | 6 | 25 | 14 | 8 | 2 | 4 | 3 | 4 |
| **89889** | 8 | 10 | 4 | 15 | 3 | 2 | 7 | 28 | 8 | 5 | 6 | 22 | 14 | 8 | 2 | 4 | 3 | 4 |
| **89936** | 8 | 10 | 4 | 11 | 3 | 2 | 7 | 16 | 8 | 5 | 6 | 16 | 15 | 8 | 2 | 4 | 3 | 4 |
| **89959** | 7 | 11 | 4 | 11 | 3 | 2 | 8 | 24 | 11 | 4 | 6 | 16 | 10 | 10 | 2 | 5 | 8 | 3 |
| **90055** | 8 | 10 | 4 | 20 | 3 | 2 | 7 | 27 | 8 | 5 | 10 | 23 | 14 | 8 | 2 | 4 | 3 | 4 |
| **90077** | 7 | 8 | 4 | 12 | 3 | 2 | 8 | 14 | 9 | 5 | 6 | 17 | 13 | 8 | 2 | 4 | 3 | 4 |
| **90148** | 8 | 10 | 4 | 20 | 3 | 2 | 7 | 29 | 8 | 5 | 7 | 15 | 14 | 8 | 2 | 4 | 3 | 4 |
| **90164** | 7 | 9 | 4 | 13 | 3 | 2 | 8 | 23 | 9 | 5 | 6 | 19 | 12 | 8 | 2 | 4 | 3 | 4 |
| **90190** | 7 | 8 | 4 | 14 | 3 | 2 | 7 | 18 | 9 | 5 | 6 | 25 | 12 | 8 | 2 | 4 | 3 | 4 |
| **90250** | 8 | 11 | 4 | 13 | 3 | 2 | 8 | 13 | 9 | 5 | 6 | 14 | 10 | 8 | 2 | 4 | 3 | 4 |
| **90305** | 7 | 9 | 4 | 15 | 3 | 2 | 9 | 25 | 9 | 5 | 6 | 13 | 16 | 8 | 2 | 5 | 3 | 4 |
| **90328** | 7 | 9 | 4 | 11 | 3 | 2 | 8 | 18 | 10 | 5 | 7 | 17 | 13 | 9 | 1 | 5 | 7 | 1 |
| **90370** | 7 | 11 | 4 | 15 | 3 | 2 | 7 | 24 | 9 | 5 | 6 | 18 | 17 | 9 | 1 | 4 | 8 | 1 |
| **90422** | 8 | 10 | 4 | 18 | 3 | 2 | 7 | 25 | 8 | 5 | 6 | 23 | 19 | 8 | 2 | 4 | 3 | 4 |
| **90469** | 8 | 11 | 4 | 11 | 3 | 2 | 7 | 28 | 8 | 5 | 6 | 29 | 14 | 8 | 2 | 4 | 3 | 4 |
| **90562** | 7 | 9 | 4 | 11 | 3 | 2 | 7 | 19 | 9 | 5 | 6 | 16 | 12 | 8 | 2 | 4 | 3 | 4 |
| **90631** | 8 | 10 | 4 | 16 | 3 | 2 | 7 | 23 | 8 | 5 | 7 | 19 | 14 | 8 | 2 | 4 | 3 | 3 |
| **90770** | 7 | 12 | 4 | 13 | 3 | 2 | 7 | 21 | 8 | 5 | 6 | 19 | 12 | 8 | 2 | 4 | 3 | 4 |
| **90961** | 6 | 10 | 4 | 14 | 3 | 2 | 8 | 13 | 8 | 6 | 6 | 19 | 21 | 11 | 1 | 4 | 7 | 1/2 |
| **91146** | 6 | 10 | 4 | 14 | 3 | ND | 7 | ND | 10 | 5 | ND | 26 | 10 | ND | 1 | ND | 3 | 1/2 |
| **91387** | 8 | 10 | 4 | 16 | 3 | 2 | 7 | 13 | 8 | 5 | 6 | 19 | 17 | 9 | 2 | 4 | 3 | 4 |
| **91600** | 7 | 9 | 4 | 12 | 3 | 2 | 8 | 21 | 9 | 5 | 6 | 26 | 11 | 9 | 2 | 4 | 3 | 4 |
| **91650** | 8 | 10 | 4 | 14 | 3 | 2 | 7 | 25 | 8 | 5 | 6 | 19 | 19 | 11 | 2 | 4 | 3 | 4 |
| **91662** | 8 | 9 | 4 | 13 | 3 | 2 | 7 | 21 | 8 | 5 | 6 | 19 | 14 | 8 | 2 | 4 | 3 | 4 |
| **91734** | 8 | 10 | 4 | 16 | 3 | 2 | 7 | 17 | 8 | 5 | 6 | 19 | 14 | 9 | 2 | 4 | 3 | 4 |
| **91758** | 7 | 10 | 4 | 11 | 3 | 2 | 8 | 15 | 8 | 5 | 6 | 26 | 12 | 8 | 2 | 4 | 3 | 4 |
| **91768** | 8 | 11 | 4 | 21 | 3 | 2 | 7 | 20 | 8 | 5 | 6 | 11 | 15 | 8 | 2 | 4 | 3 | 4 |
| **91796** | 7 | 9 | 4 | 13 | 3 | 2 | 8 | 16 | 9 | 5 | 6 | 21 | 11 | 8 | 2 | 4 | 3 | 4 |
| **91840** | 7 | 12 | 4 | 11 | 3 | 2 | 7 | 20 | 10 | 4 | 6 | 12 | 13 | ND | 2 | 5 | 9 | 3 |
| **91845** | 8 | 9 | 4 | 17 | 3 | 2 | 8 | 16 | 9 | 5 | 6 | 23 | 15 | 8 | 2 | 4 | 3 | 4 |
| **91884** | 7 | 9 | 4 | 12 | 3 | 2 | 8 | 14 | 9 | 5 | 6 | 18 | 12 | 8 | 2 | 4 | 3 | 4 |
| **91916** | 8 | 10 | 4 | 13 | 3 | 2 | 7 | 17 | 8 | 5 | 6 | 19 | 14 | 8 | 2 | 4 | 3 | 4 |
| **91928** | 7 | 8 | 4 | 10 | 3 | 2 | 7 | 14 | 9 | 5 | 6 | 17 | 15 | 8 | 2 | 4 | 3 | 4 |
| **91979** | 7 | 9 | 4 | 13 | 3 | 2 | 7 | 16 | 9 | 5 | 6 | 14 | 11 | 8 | 2 | 4 | 3 | 4 |
| **92008** | 7 | 9 | 4 | 14 | 3 | 2 | 8 | 19 | 9 | 5 | 6 | 22 | 12 | 8 | 2 | 4 | 3 | 4 |
| **92032** | 8 | 11 | 4 | 13 | 3 | 2 | 7 | 19 | 8 | 5 | 6 | 17 | 13 | 9 | 2 | 4 | 3 | 4 |
| **92070** | 7 | 10 | 4 | 14 | 3 | 2 | 9 | 16 | 11 | 5 | 6 | 26 | 9 | 8 | 2 | 4 | 3 | 4 |
| **92114** | 8 | 9 | 4 | 14 | 3 | 2 | 7 | 25 | 8 | 5 | 6 | 20 | 15 | 9 | 2 | 4 | 3 | 4 |
| **92278** | 7 | 9 | 4 | 10 | 3 | 2 | 7 | 26 | 9 | 5 | 6 | 17 | 14 | 8 | 2 | 4 | 3 | 4 |
| **92367** | 7 | 12 | 4 | 13 | 3 | 2 | 7 | 23 | 8 | 5 | 6 | 20 | 13 | 8 | 2 | 4 | 3 | 4 |
| **92409** | 8 | 12 | 4 | 13 | 3 | 2 | 7 | 22 | 8 | 5 | 8 | 24 | 13 | 8 | 2 | 5 | 3 | 4 |
| **92479** | 7 | 8 | 4 | 13 | 3 | 2 | 8 | 20 | 8 | 5 | 6 | 16 | 13 | 11 | 1 | 5 | 3 | 1/2 |
| **92500** | 8 | 10 | 4 | 14 | 3 | 2 | 7 | 19 | 8 | 5 | 6 | 22 | 17 | 8 | 2 | 4 | 3 | 4 |
| **92577** | 7 | 8 | 4 | 13 | 3 | 2 | 8 | 22 | 10 | 5 | 6 | 17 | 13 | 13 | 1 | 5 | 8 | 3 |
| **92626** | 7 | 7 | 4 | 13 | 3 | 2 | 8 | ND | 8 | 5 | 6 | ND | ND | ND | 2 | ND | ND | 4 |
| **92642** | 8 | 11 | 4 | 17 | 3 | 2 | 8 | 19 | 8 | 5 | 6 | 17 | 13 | 8 | 2 | 4 | 3 | 4 |
| **92743** | 8 | 8 | 4 | 13 | 3 | 2 | 8 | 20 | 8 | 5 | 6 | 26 | 13 | 12 | 1 | 5 | 3 | 3 |
| **92823** | 8 | 10 | 4 | 16 | 3 | 2 | 7 | 14 | 8 | 5 | 6 | 15 | 15 | 8 | 2 | 4 | 3 | 4 |
| **92873** | 7 | 8 | 4 | 13 | 3 | 2 | 7 | 17 | 8 | 5 | 6 | 26 | 14 | ND | 2 | 5 | 8 | 3 |
| **92989** | 7 | 12 | 4 | 13 | 3 | 2 | 7 | 22 | 8 | 5 | 6 | 17 | 11 | 8 | 2 | 4 | 3 | 4 |
| **93045** | 7 | 9 | 4 | 10 | 3 | 2 | 8 | 14 | 9 | 5 | 6 | 26 | 13 | 8 | 2 | 4 | 3 | 4 |
| **93089** | 7 | 9 | 4 | 13 | 3 | 2 | 8 | 18 | 10 | 5 | 7 | 18 | 13 | 9 | 1 | 5 | 7 | 1/2 |
| **93093** | 7 | 9 | 4 | 13 | 3 | 2 | 8 | ND | 9 | 5 | 6 | 26 | 11 | 8 | 2 | 4 | 3 | 4 |
| **93145** | 7 | 9 | 4 | 16 | 3 | 2 | 7 | 13 | 9 | 5 | 6 | 17 | 14 | 9 | 1 | 4 | 8 | 1/2 |
| **93321** | 8 | 10 | 4 | 18 | 3 | 2 | 7 | 19 | 8 | 5 | 6 | 18 | 16 | 8 | 2 | 4 | 3 | 4 |
| **93354** | 8 | 11 | 4 | 12 | 3 | 2 | 7 | 14 | 10 | 5 | 6 | 17 | 13 | 8 | 2 | 3 | 3 | 4 |
| **93388** | 8 | 11 | 4 | 13 | 3 | 2 | 7 | ND | 8 | 5 | 6 | 26 | 14 | 8 | 2 | 4 | 3 | 3 |
| **93389** | 8 | 10 | 4 | 20 | 3 | 2 | 7 | 26 | 8 | 5 | 6 | 16 | 16 | 8 | 2 | 4 | 3 | 4 |
| **93400** | 8 | 10 | 4 | 16 | 3 | 2 | 7 | 30/31 | 8 | 5 | 6 | 25 | 13 | 8 | 2 | 4 | 3 | 4 |
| **93467** | 8 | 11 | 4 | 23 | 3 | 2 | 7 | 20 | 8 | 5 | 6 | 11 | 15 | 8 | 2 | 4 | 3 | 4 |
| **93703** | 7 | 12 | 4 | 13 | 3 | 2 | 7 | 23 | 8 | 5 | 6 | 21 | 10 | 8 | 2 | 4 | 3 | 4 |
| **93706** | 8 | 12 | 4 | 14 | 3 | 2 | 7 | 22 | 8 | 5 | 6 | 12 | 14 | 8 | 2 | 4 | 3 | 4 |
| **94004** | 7 | 8 | 4 | 14 | 3 | 2 | 8 | 20 | 10 | 5 | 6 | 17 | 13 | 10 | 1 | 5 | 8 | 1/2 |

ND = not done
