# Supplementary material for: Genotyping of Mycobacterium leprae for better understanding of leprosy transmission in Fortaleza, Northeastern Brazil
Source: PLoS Negl Trop Dis. 2017 Dec 15;11(12):e0006117. doi: 10.1371/journal.pntd.0006117 (PMC5747459; doi:10.1371/journal.pntd.0006117)
Supplement: S2 Table — (DOC) [file pntd.0006117.s007.doc]

**S2 Table.** Bivariate analysis of demographic, socioeconomic, behavioral, and environmental variables with leprosy.

**Variables Non clustered* Clustered* p value**

**Nr (%/mean value) Nr (%/mean value)**

**Clinical**

Number of months between observing the first lesion and diagnosis/notification

2.1 (40.3) 5.5 (59.7) 0.14

Number of lesions 59 (16.1) 86 (13.6) 0.28

Number of affected nerves 46 (1.5) 62 (1.3) 0.55

Number of contacts 32 (3.3) 49 (3.4) 0.73

Bacillary index 55 (3.1) 78 (3.2) 0.72

Acid Fast Bacilly

Pos 30 (49.2%) 60(31%) **0.037**

Neg 29 (50.8%) 27 (69%)

Disability

Grade 0 26 (66.7%) 38 (62.3%) 0.43

Grade 1 11 (28.2%) 15 (24.6%)

Grade 2 2 (5.1%) 8 (13.1%)

Clinical form

Borderline 36 (58.1%) 51 (56.0%) 0.81

Tuberculoid 1 (1.6%) 3 (3.3%)

Lepromatous 25 (40.3%) 37 (40.7%)

BCG taken

Yes 35 (74.5%) 54 (75.0%) 0.43

No 5 (10.6%) 14 (19.4%)

*No info? 7 (14.9%) 4 (5.6%)***

BCG scar

Yes 32 (51.6%) 41 (43.6%) 0.16

No 20 (32.3%) 45 (47.9%)

*No info 10 (16.1%) 8 (8.5%)**

**Socioeconomic variables**

Schooling

None/1st grade incomplete 24 (58.5%) 32 (56.1%) 0.60

1^st^ grade complete/2^nd^ incomplete 9 (22%) 17 (29.8%)

2^nd^ complete/superior/post 8 (19.5%) 8 (14%)

Family income

Less than one minimum salary 5 (12.2%) 11 (19.6%) 0.42

More than one minimum salary 36 (87.8%) 45 (80.4%)

Instruction level of the head of the family?

Analphabet/incomplete 1^st^ degree 31 (77.5%) 35 (64.8%) 0.41

1^st^complete/2^nd^ incomplete 6 (15%) 13 (24.1%)

2^nd^ complete/superior 3 (7.5%) 6 (11.1%)

Socio-economic level

C 12 (29.3%) 14 (24.6%) 0.65

D/E 29 (70.7%) 43 (75.4%)

Food shortage for the family at any time of life

Yes 31 (75.6%) 48 (84.2%) 0.29

No 10 (24.4%) 8 (14%)

Did you experience serious financial problems during your life?

Yes 31 (75.6%) 44 (78.6%) 0.80

No 10 (24.4%) 12 (21.4%)

Do you smoke

Yes 7 (17.1%) 9 (15.8%) 1

No 34 (82.9%) 48 (84.2%)

Alcohol use

Never 24 (58.5%) 44 (77.2%) **0.047**

Max once a week 12 (29.2%) 12 (21.1%)

Several times a week 5 (12.2%) 1 (1.8%)

**Environmental variables**

Number of persons in your residence 41 (3.98) 57 (4.49) 0.29

How many people with leprosy you know 2.1 (48.9%) 1.44 (51.1%) 0.13

How many months ago was first contact? 21 (240) 17 (157) 0.24

Is the contact a parent with frequent contact at home?

Yes 12 (57.1%) 16 (40.7%) 0.78

No 9 (57.1%) 16 (59.3%)

Has this person lived or lives with you?

Yes 8 (36.4%) 3 (13%) 0.09

No 14 (63.6%) 20 (87%)

Did you/the contact sleep in the same bed/hammock?

Yes 4 (18.2%) 1 (4.3%) 0.19

No 18 (81.8%) 22 (95.7%)

Did you sleep in the same room?

Yes 5 (22.7%) 3 (13%) 0.46

No 17 (77.3%) 20 (87%)

Did you use the same clothes?

No 22 (100%) 23 (100%)

Did you use the same towel?

Yes 4 (18.2%) 1 (4.3%) 0.19

No 18 (81.8%) 22 (92.7%)

Were lovers?

Yes 2 (9.1%) 0 0.23

No 20 (90.9%) 23 (100%)

Is the contact your neighbor?

Yes 4 (19%) 3 (11.1%) 0.68

No 17 (81%) 24 (88.9%)

Is it presently a contact at work?

Yes 0 3 (11.1%) 0.25

No 21 (100%) 24 (88.9%)

Has it been a work contact in the past?

Yes 0 5 (21.7%) 0.049***

No 22 (100%) 18 (78.3%)

How do you think you got leprosy

Don’t know 24 (58.5%) 39 (68.4%) 0.41

Close family 8 (19.5%) 6 (10.5%)

Neighbor/work 3 (7.3%) 2 (3.5%)

Friends 3 (7.3%) 3 (5.3%)

River/beach/mud 0 2 (3.5%)

*Others 0 4 (7%)**

Have you heard about leprosy before your diagnosis?

Yes 33 (80.5%) 41 (71.9%) 0.35

No 8 (19.5%) 16 (28.1%)

**Demographic variables**

Sex

Male 41 (66.1%) 66 (70.2%) 0.60

Female 21 (33.9%) 28 (29.8%)

Age 62 (42.1) 94 (41.8) 0.95

Skin color

White 16 (27.1%) 18 (20%) 0.73

Brown 36 (61%) 62 (68.9%)

Black 5 (8.5%) 8 (8.9%)

Yellow 2 (3.4%) 2 (2.2%)

White/yellow 18 (30.5%) 20 (22.25%) 0.34

Black/Brown 41 (69.5%) 70 (77.8%)

Civil state

Free 12 (29.3%) 18 (31.6%) 0.99

Married/living together 23 (56.1%) 30 (52.6%)

Divorced 4 (9.8%) 6 (10.5%)

Widow 2 (4.9%) 3 (5.3%)

98 of the individuals had data generated as part of the project with prospective data; the other 61 (38.4%) had their data retrieved from the SINAN ([http://sinan.saude.gov.br](http://sinan.saude.gov.br/))

*Clustering defined by excluding the four most variable VNTRs

**not included for chi-square calculation

***dependent variables
