# Supplementary material for: Genotyping of Mycobacterium leprae for better understanding of leprosy transmission in Fortaleza, Northeastern Brazil
Source: PLoS Negl Trop Dis. 2017 Dec 15;11(12):e0006117. doi: 10.1371/journal.pntd.0006117 (PMC5747459; doi:10.1371/journal.pntd.0006117)
Supplement: S3 Table — (DOC) [file pntd.0006117.s008.doc]

**S3 Table.** Increase in clustering by inclusion of genotypes of *Mycobacterium leprae* present in nasal swabs.

| **Sample** | **Genetic marker** | | | | | | | | | | | |
| --- | --- | --- | --- | --- | --- | --- | --- | --- | --- | --- | --- | --- |
|  | **(AC)8b** | **(GTA)9** | **(GGT)5** | **21-3** | **(AC)9** | **(AC)8a** | **27-5** | **6-7** | **(TA)10** | **23-3** | **12-5** | **18-8** |
| **85886B*** | 7 | 9 | 4 | 2 | 8 | 9 | 5 | 7 | 8 | 2 | 4 | 3 |
| **91796N*** | 7 | 9 | 4 | 2 | 8 | 9 | 5 | 7 | 8 | 2 | 4 | ND** |
| **86187N** | 8 | 10 | 4 | 2 | 7 | 8 | 5 | 6 | NA*** | 2 | 4 | ND |
| **87401N** | 8 | 10 | 4 | 2 | 7 | 8 | 5 | 6 | NA | 2 | 4 | ND |
| **87126B** | 8 | 10 | 4 | 2 | 7 | 8 | 5 | 7 | 8 | 2 | 4 | 3 |
| **87250B** | 8 | 10 | 4 | 2 | 7 | 8 | 5 | 7 | 8 | 2 | 4 | 3 |
| **88737B** | 8 | 10 | 4 | 2 | 7 | 8 | 5 | 7 | 8 | 2 | 4 | 3 |
| **90148B** | 8 | 10 | 4 | 2 | 7 | 8 | 5 | 7 | 8 | 2 | 4 | 3 |
| **90631B** | 8 | 10 | 4 | 2 | 7 | 8 | 5 | 7 | 8 | 2 | 4 | 3 |
| **88689N** | 8 | 10 | 4 | 2 | 7 | 8 | 5 | 7 | 8 | 2 | 4 | ND |
| **86855N** | 8 | 10 | 4 | 2 | 7 | 8 | 5 | 7 | NA | 2 | 4 | ND |
| **87126N** | 8 | 10 | 4 | 2 | 7 | 8 | 5 | 7 | NA | 2 | 4 | ND |
| **86855B** | 8 | 10 | 4 | 2 | 7 | 9 | 5 | 7 | 8 | 2 | 4 | 3 |
| **90148N** | 8 | 10 | 4 | 2 | 7 | 9 | 5 | 7 | 8 | 2 | 4 | ND |
| **91979N** | 8 | 10 | 4 | 2 | 7 | 9 | 5 | 7 | 8 | 2 | 4 | ND |

*N = nasal swab; B = skin biopsy; ** ND = not done; *** NA = no amplification
