# Supplementary material for: Genotyping of Mycobacterium leprae for better understanding of leprosy transmission in Fortaleza, Northeastern Brazil
Source: PLoS Negl Trop Dis. 2017 Dec 15;11(12):e0006117. doi: 10.1371/journal.pntd.0006117 (PMC5747459; doi:10.1371/journal.pntd.0006117)
Supplement: S4 Table — (DOC) [file pntd.0006117.s009.doc]

**S4 Table.** Distribution of genotyped cases among the neighborhoods of Fortaleza.

**Cluster 0 1 2 3 4 5 6 7 8 9 10 11 12 13 14 15 16 17 18 19 20 Total**

**Cases** 62 3 2 2 5 2 2 4 3 3 2 2 19 2 23 3 5 6 2 2 2 **156**

**Neighborhoods*** 7 3 1 2 5 2 2 2 2 2 2 2 17 2 18 3 4 5 2 1 2 **86**

**Other Municipality** 2 0 0 0 0 0 0 0 0 1 0 0 0 0 0 0 1 1 0 0 0 **5**

*In Fortaleza
